# Cx43 Present at the Leading Edge Membrane Governs Promigratory Effects of Osteoblast-Conditioned Medium on Human Prostate Cancer Cells in the Context of Bone Metastasis

**DOI:** 10.3390/cancers12103013

**Published:** 2020-10-16

**Authors:** Jonathan Boucher, Annie-Claire Balandre, Marjolaine Debant, Justine Vix, Thomas Harnois, Nicolas Bourmeyster, Elodie Péraudeau, Amandine Chépied, Jonathan Clarhaut, Françoise Debiais, Arnaud Monvoisin, Laurent Cronier

**Affiliations:** 1 CNRS ERL7003, Laboratory Signalisation et Transports Ioniques Membranaires (STIM), University of Poitiers, 1 rue Georges Bonnet, TSA 51106, CEDEX 09, 86073 Poitiers, France; jonathan.boucher@univ-poitiers.fr (J.B.); annie-claire.balandre@univ-poitiers.fr (A.-C.B.); m.debant@leeds.ac.uk (M.D.); justine.vix@chu-poitiers.fr (J.V.); thomas.harnois@univ-poitiers.fr (T.H.); nicolas.bourmeyster@univ-poitiers.fr (N.B.); francoise.debiais@chu-poitiers.fr (F.D.); arnaud.monvoisin@univ-poitiers.fr (A.M.); 2 Department of Rheumatology, University Hospital Center of Poitiers, 2 Rue de la Milétrie, 86021 Poitiers, France; 3 University Hospital Center of Poitiers, 2 rue de la Milétrie, 86021 Poitiers, France; elodie.peraudeau@univ-poitiers.fr (E.P.); jonathan.clarhaut@univ-poitiers.fr (J.C.); 4 CNRS UMR 7285, Institut de Chimie des Milieux et des Matériaux de Poitiers (IC2MP), University of Poitiers, 4 Rue Michel Brunet, TSA 51106, CEDEX 09, 86073 Poitiers, France; 5 Laboratory of Experimental and Clinical Neurosciences, LNEC-INSERM U1084, UBM-Laboratoire de Cancérologie Biologique, CHU de Poitiers, 2 Rue de la Milétrie, 86000 Poitiers, France; amandine.chepied@univ-poitiers.fr

**Keywords:** Cx43, prostate cancer, migration, gap junctions, bone metastasis, conditioned medium, carboxy terminal domain

## Abstract

**Simple Summary:**

In its late stages, prostate cancer (PCa) is characterized by a high propensity to form osteoblastic bone metastases, mainly treated by palliative approaches. In a previous work, we demonstrated that a gap junctional protein, connexin43 (Cx43) is implicated both in the increase of aggressiveness of PCa cells and in their impact on bone. To analyze the reciprocal part of the dialogue, the current study addresses the role of Cx43 in the impact of bone microenvironment on PCa cells abilities. Using Cx43-overexpressing PCa cell lines, we determined that Cx43 is necessary for promigratory effect induced by osteoblastic conditioned media (ObCM) on individual cells. Next, we demonstrated the requirement of Cx43 membrane localization at the leading edge and the involvement of the cytoplasmic part in this ObCM-induced migration. Overall, our findings precise the role of Cx43 during PCa progression and its putative use as aggressiveness marker and as potential therapeutic targets.

**Abstract:**

Among the different interacting molecules implicated in bone metastases, connexin43 (Cx43) may increase sensitivity of prostate cancer (PCa) cells to bone microenvironment, as suggested by our in silico and human tissue samples analyses that revealed increased level of Cx43 expression with PCa progression and a Cx43 specific expression in bone secondary sites. The goal of the present study was to understand how Cx43 influences PCa cells sensitivity and aggressiveness to bone microenvironment. By means of Cx43-overexpressing PCa cell lines, we revealed a Cx43-dependent promigratory effect of osteoblastic conditioned media (ObCM). This effect on directional migration relied on the presence of Cx43 at the plasma membrane and not on gap junctional intercellular communication and hemichannel functions. ObCM stimulation induced Rac1 activation and Cx43 interaction with cortactin in protrusions of migrating PCa cells. Finally, by transfecting two different truncated forms of Cx43 in LNCaP cells, we determined that the carboxy terminal (CT) part of Cx43 is crucial for the responsiveness of PCa cells to ObCM. Our study demonstrates that Cx43 level and its membrane localization modulate the phenotypic response of PCa cells to osteoblastic microenvironment and that its CT domain plays a pivotal role.

## 1. Introduction

Prostate cancer (PCa) is the most common male cancer in term of worldwide incidence and represents one of the major causes from cancer death in men (World Health Organization, Geneva, Switzerland; Globocan 2018, estimated age-standardized rates, all ages). This androgen-sensitive cancer is characterized by a significant rise in case number with age and a distinct feature of human PCa is the net propensity to disseminate towards bone and to develop metastasis. Bone metastases, present in more than 70% of prostate cancer patients in the advanced stages, are associated with dramatic complications mainly skeletal-related events (SRE) linked to both osteogenesis and osteolysis [1]. To date, only palliative treatments for these bone metastases and androgen-deprivation therapies with high potential side effects are mainly available. Moreover, the prediction of PCa cells aggressiveness based on biomarkers to distinguish highly aggressive cells remains challenging due to the well-described histological and cellular heterogeneity of this multifocal cancer [2].

The bone privileged metastatic site is particularly due to the vicious dialogue between the cancer cell and the bone microenvironment present in two coexisting niches, namely endosteal and hematopoietic niches. Indeed, soluble factors, cell surface proteins, matrix-associated components and cancer-associated cells exhibit pivotal roles in the regulation of proliferation, survival, adhesion, dormancy and migration of disseminating PCa cells in the skeletal context. These factors facilitate the survival and adaptation of PCa cells to their new environment. In addition to these extensively investigated aspects, components of direct cell-cell interactions were also postulated to play a role in the aggressiveness and dissemination of cancer cells. Among them, intercellular channels composed of connexins (Cx) leading to a gap junction intercellular communication (GJIC) between cytoplasm of adjacent cells were soundly involved [3,4].

Because of their unique function in tissue homeostasis by GJIC, a major role of Cxs loss in the onset and the development of cancer was historically demonstrated [4]. These transmembrane proteins belong to a multigene family of 21 members in human and share a common primary structure with four transmembrane and two extracellular domains and three cytosolic portions corresponding to N-term, intracellular loop and a long C-term part [5]. This latter domain also called carboxy terminal (CT) domain accounts for the difference between Cxs, notably in regulatory processes with potential phosphorylation and interaction sites. In addition to the well-known GJIC linked to formation of intercellular channels, recent data demonstrated two non-canonical alternative functions for Cxs [6,7]. Hemichannels or connexons, formed by hexamerization of Cxs outside the junctional plaques, induce a communication with the extracellular space [8]. The CT domain is considered as a controller of signaling networks inside the cells via an “interactome” function [9]. Although each connexin isoform exhibits a distinct tissue distribution, many cell types express more than one connexin. In normal prostatic epithelium, experimental evidences reported the presence of 3 distinct Cx subtypes, Cx32, Cx43, and Cx26, encoding by genes named GJB1, GJA1, and GJB2, respectively (for review, see Reference [10]).

During cancer progression, an undeniable but ambivalent role was demonstrated for Cx43 in different types of carcinomas. For many years, Cx43 was considered as a tumor suppressor with accumulating evidences demonstrating anti-proliferative effects in most of cancer cells. Thus, Connexin 43 expression was significantly reduced or lost in prostate cancer tissues from patients with clinically localized prostate cancer [11]. In addition to the modulation of Cx43 expression level, an alteration of the traffic to the plasma membrane was also demonstrated especially in androgen-insensitive PCa cells [12]. However, more recent data highlighted a pro-aggressive action of Cx43 during most of dissemination steps leading to colonization of secondary sites [6,13]. During prostate cancer progression, the intrinsic promigratory and proinvasive phenotype of PCa cells was clearly linked to an increase in Cx43 expression [10,14,15,16]. Thus, Cx43 expression level in PCa cells was correlated with transcription factors and proteolytic activities implicated in the epithelial-mesenchymal transition [15,17]. During diapedesis or angiogenesis, Cx43 has been shown to facilitate heterocellular dialogue with endothelial cells [18,19]. Moreover, increasing number of publications have shown that Cx43 is implicated in the control of cell migration during cancer dissemination process [14,20,21].

In the bone metastatic context, our previous data using Cx43-overexpressing PCa cell lines clearly demonstrated in vitro and in vivo that Cx43 enhanced the metastatic behavior of tumor cells [15]. This increased aggressiveness was linked to Cx43 expression level and was restricted to cellular model presenting a functional exportation to the plasma membrane. The impact on bone integrity was due to both alteration of proliferation rate and differentiation abilities of the bone-forming cells (osteoblasts) leading to an osteolytic impact through both GJIC-independent and -dependent actions. More recently, Wang et al. (2018), reported that osteoblastic cells in the osteogenic niche could serve as calcium reservoir transferable by GJIC to cancer cells and could promote bone metastatic progression [22].

To date, only a limited number of controverted studies have analyzed the significance of Cx43 expression level during PCa progression in human samples [11,23,24]. No immunohistochemical study performed on bone metastatic tissues really focused on Cx43 expression. Moreover, the mechanisms underlying the implication of Cx43 in the chemotaxis of PCa cells to bone tissue and in the development of bone metastasis are incompletely understood. In the context of a crosstalk established during the formation of bone metastasis, the present work focused on the roles played by Cx43 expression level and its localization in the sensitivity of PCa cells to bone microenvironment.

In this study, we demonstrate that Cx43 potentiates the migration ability of PCa cells and the sensitivity to ObCM. We found that Cx43 membrane localization at the leading edge is required for this promigratory effect and that the carboxyl-terminal domain of Cx43, which interacts with Rac1 and cortactin, is sufficient to sustain cell migration in this bone context. Our data strengthen the protumoral propensity of the carboxy terminal domain of Cx43 and demonstrate that, in the metastatic context, it can modulate the phenotypic response of PCa cells to the osteoblastic environment.

## 2. Results

### 2.1. Cx43 Expression Varies with PCa Progression and Exhibits a Specific Pattern in Bone Secondary Sites

**Figure 1:**
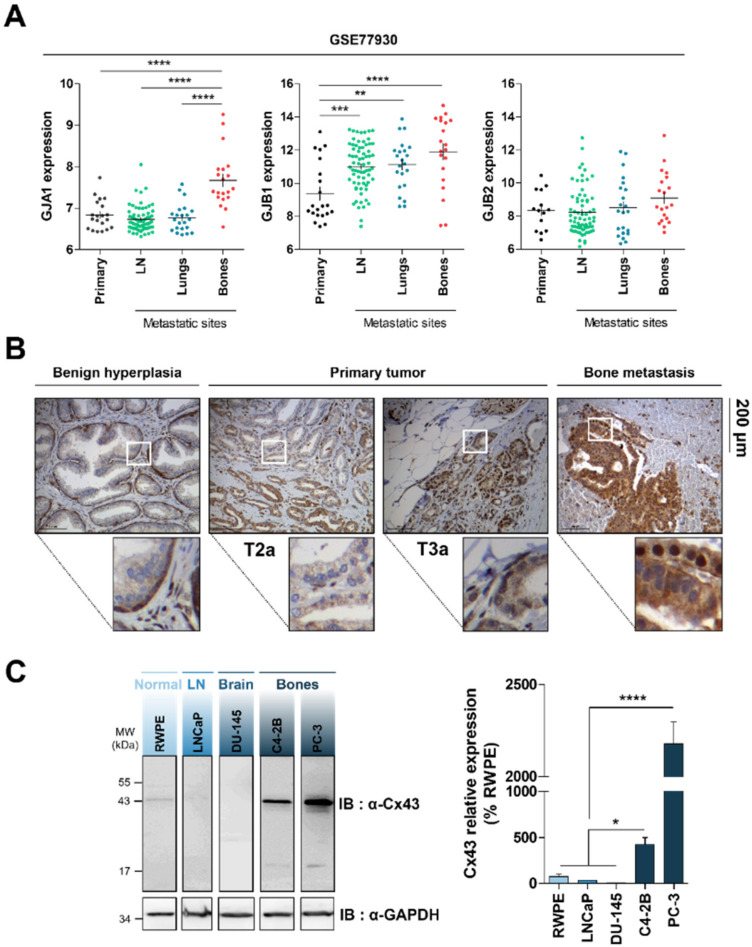
GJA1 gene and protein expression levels are associated with prostate cancer aggressiveness and bone metastases. (**A**) GJA1, GJB1, and GJB2 expression levels extracted from dataset GSE77930 were compared in 130 Castration-resistant Prostate Cancer (CRPC) tumors samples between primary tumor (*n* = 20) and metastatic sites including lymph nodes (*n* = 68), lungs (*n* = 22), and bones (*n* = 20). Data represent the mean ± SEM from indicated number of samples. (**B**) Immunohistochemical staining of connexin43 (Cx43) in tumor samples from patients with different grades of prostate cancer, including T2a (*n* = 5) and T3a (*n* = 5) from primary tumors and bone metastasis (*n* = 10) compared to non-malignant Benign Prostatic Hyperplasia (*n* = 5). (**C**) Representative immunoblot and densitometric quantification of Cx43 in 4 different prostate cancer cell lines (LNCaP, DU-145, C4-2B, PC-3) in comparison with normal prostatic epithelial cell line (RWPE). GAPDH served as loading control. Data represent the mean ± SEM from 3 different experiments. * p < 0.05; ** p < 0.01; *** p < 0.001; **** p < 0.0001.

To test the hypothesis of a specific connexin profile in bone metastatic cells of prostate origin, we performed a comparison of GJA1, GJB1, and GJB2 expression levels between bone and other metastatic sites of PCa from a published dataset (GEO: GSE77930) [2]. In this dataset, custom Agilent 44K whole human genome expression oligonucleotide microarrays were used to profile 171 Castration-Resistant PCa tumors from 63 patients and RNA was amplified prior to hybridization against a common reference pool of prostate tumor cell lines (LNCaP, DU145, PC3, CWR22). Our in silico analysis revealed a significant up-regulation of GJA1 encoding Cx43 in bone metastatic lesions (Figure 1A) along other genes previously associated with bone tropism (Supplementary Figure S1A), compared to primary tumors and to two other metastatic sites (lymph nodes and lungs). This differential expression was not observed for two other common Cx genes expressed in the normal prostatic tissue, namely, GJB1 (Cx32) and GJB2 (Cx26) (Figure 1A). Similar results were also obtained with another published set (GEO: GSE32269) [25], showing a specific increase of Cx43 expression in bone metastases compared to the primary site (Figure S1B).

To validate the correlation between GJA1 expression and PCa grade, immunohistochemical staining of Cx43 was performed on tissue from 5 benign prostatic hyperplasia, 5 PCa of grade 2 and grade 3 and 10 of bone metastasis specimen (Figure 1B). The same experiment was done using a prostate disease spectrum tissue microarray (PR8011b, US Biomax Inc., Rockville, MD, USA), containing cases of adenocarcinoma, metastatic carcinoma, hyperplasia tissue, adjacent and normal prostate tissue (Figure S1C). As previously shown, a strong immunoreactivity was revealed in basal prostatic cells in normal and hypertrophic prostate with a characteristic punctuate membranous and intracytoplasmic staining (Figure 1B and Figure S1C). In grade 2 and 3 tumors, analysis of the pattern of Cx43 expression was more complex with a noted variability in immunodetection between biopsies and inside biopsies illustrating the multifocal feature of primary prostate cancer [26]. However, in most cases, Cx43 expression was dramatically reduced or absent in grade 2 and 3 tumors, whereas a strong staining was present in grade 4 tumors and bone metastatic cells (Figure 1B and Figure S1C). In these later stages, Cx43 exhibited an increased cytoplasmic and nuclear immunostaining compared to normal and precocious stages.

We then analyzed the Cx43 expression level by Western blotting in normal prostatic epithelial cells (RWPE) and in 4 different cancer cell lines typically used for in vitro studies (Figure 1C). As previously reported [14], Cx43 expression was associated with the degree of malignancy leading to a reduced expression in low aggressive model (LNCaP) compared to RWPE. Interestingly, we also revealed a progressive increase of Cx43 protein level with the propensity to colonize bone sites, whereas no gain was demonstrated in metastatic cells targeting central nervous system (DU145). These data suggest that Cx43 is specifically linked to the dissemination of prostatic cancer cells towards bones.

### 2.2. Cx43 Enhances the Migration Ability of LNCaP Cells and the Sensitivity to Bone Conditioned Medium

**Figure 2:**
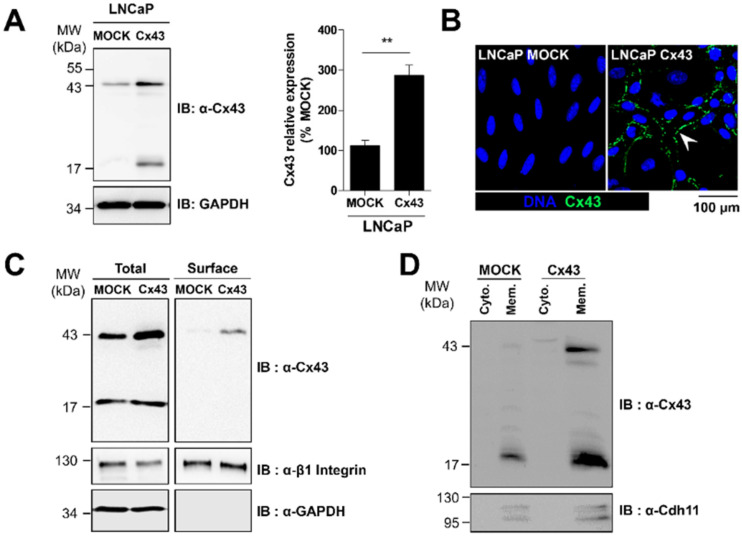
Characterization of ectopic Cx43 expression at the plasma membrane in LNCaP cells. (**A**) Representative immunoblot and densitometric quantification of Cx43 in LNCaP cells stably transfected to overexpress Cx43. Data represent the mean ± SEM from 7 different experiments. (**B**) Immunostaining of Cx43 (green) in LNCaP MOCK and LNCaP Cx43 showing positive staining at cell-cell contacts (white arrow head). Nuclei were DAPI-stained (blue). (**C**) Surface biotinylation assay and associated immunoblot showing a specific Cx43 enrichment at the surface of LNCaP Cx43 cells. Total cell surface proteins (immunoprecipitated from streptavidin beads) LNCaP MOCK and Cx43 were immunoblotted with indicated antibodies. Integrin β1 and GAPDH served, respectively, as positive and negative control of cell surface expression. (**D**) Isolation of membrane proteins and associated immunoblot analysis showing Cx43 enrichment in membrane fraction. Cytosolic and membrane protein extracts from LNCaP MOCK and Cx43 were immunoblotted with indicated antibodies. Cadherin 11 served as a positive control for membrane enriched fraction. ** p < 0.01.

In order to investigate the potential role of Cx43 in bone dissemination of PCa cells, we used Cx43-overexpressing low aggressive LNCaP cells [15]. The stable increase of Cx43 by 2.6-fold over the endogenous expression in the total population (Figure 2A) resulted in a preferential membrane expression of Cx43 as revealed by immunofluorescence staining (Figure 2B) and confirmed by cell-surface biotinylation and subcellular fractionation assays (Figure 2C,D). Interestingly, the monoclonal antibody directed against the C-terminus, was also efficient to detect a shorter isoform at 20 kDa (GJA1-20k), both in cell lysates and whole-membrane fractions. This isoform was recently described in different cancer cell lines [27].

The full length Cx43 localization leads to functional gap junctional intercellular communication (GJIC) as endorsed by preloading experiments (Figure S2) and previously validated by Fluorescence Recovery After Photobleaching (FRAP) experiments in Reference [15]. To test the possible influence of Cx43 in the bone microenvironment-dependent sensitivity of PCa cells, we next evaluated the effects of osteoblastic-conditioned medium (ObCM) on the main phenotypic characteristics of PCa cells.

Firstly, in terms of proliferation, we demonstrated that neither isolated osteoblastic cells or ObCM, nor explants of total calvaria could modulate the proliferative potential of the LNCaP cells, regardless of their Cx43 expression level (Figure S3A–C). To address whether Cx43 overexpression in LNCaP affects apoptosis, cell death was determined using the annexin-V and Propidium Iodide staining followed by flow cytometry analysis. Spontaneous apoptosis was not significantly different in our culture conditions between LNCaP transfected or not with Cx43 (9.36 ± 2.99% and 7.44 ± 1.98%, respectively). Moreover, we found that ObCM stimulation for 17 h did not significantly affect apoptosis of LNCaP cells overexpressing or not Cx43 (Figure S3D).

**Figure 3:**
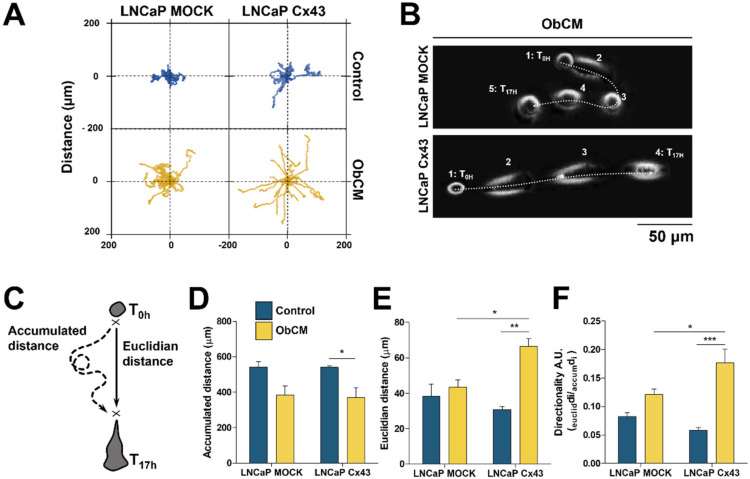
Cx43 induces osteoblasts-dependent migration of LNCaP cells. Quantitative analysis of single cell migration of LNCaP using time-lapse videomicroscopy. Cells were incubated for 17 h with either control or osteoblasts-conditioned media (ObCM), and records were used for manual tracking of cell centroid trajectories. (**A**) Scaled spider graph showing for each condition the individual paths of 15 representative cells starting from their initial position at T = 0. (**B**) Overlapped phase contrast images showing the shape and the trajectory over time of representative LNCaP MOCK and Cx43 cells upon ObCM stimulation. (**C**) Three dynamic parameters were quantified, the total path length, referred to as accumulated distance (μm) (**D**) the straight distance between the starting and ending point of the trajectory referred to as ‘Euclidian distance’ (μm) (**E**) and the directionality defined by the accumulated distance divided by the Euclidian distance (**F**). Data represent the mean ± SEM from 4 different experiments (MOCK CTRL *n* = 167; MOCK ObCM *n* = 179; Cx43 CTRL *n* = 171; Cx43 ObCM *n* = 166). * p < 0.05; ** p < 0.01; *** p < 0.001.

As migration and invasion capacities represent important features of PCa cells homing to bone, we thought to measure whether tumoral Cx43 expression level could affect both of these processes upon osteoblastic stimulation. We therefore evaluated the invasion abilities of LNCaP cells transfected with Cx43, stimulated or not by differentiating osteoblastic cells (Figure S4). We found that under control conditions, Cx43 overexpression did not modulate LNCaP cells invasion as compared with mock cells. Interestingly, we observed a 4-fold increase of cell invasion induced by the co-culture with osteoblasts independently of the Cx43 level. Finally, to determine the impact of both Cx43 overexpression and bone microenvironment on the migration of LNCaP cells, we carried out time-lapse microscopy of single cells migrating for 17 h (Figure 3). We demonstrated that overexpression of Cx43 had by itself no effect on LNCaP cells migration in control conditions. However, stimulation with ObCM significantly induced directional cell movement of LNCaP cells overexpressing Cx43 cells as compared with mock cells (Figure 3A). Analyses of dynamic parameters of this single cell motility allowed us to demonstrate a significant increase in Euclidian distance and in directionality for LNCaP-Cx43 in the presence of conditioned medium, whereas the accumulated distance was reduced (Figure 3B,F). Taken together, these data suggest that Cx43 is involved in the polarization and migration of LNCaP cells under bone microenvironment stimuli.

**Figure 4:**
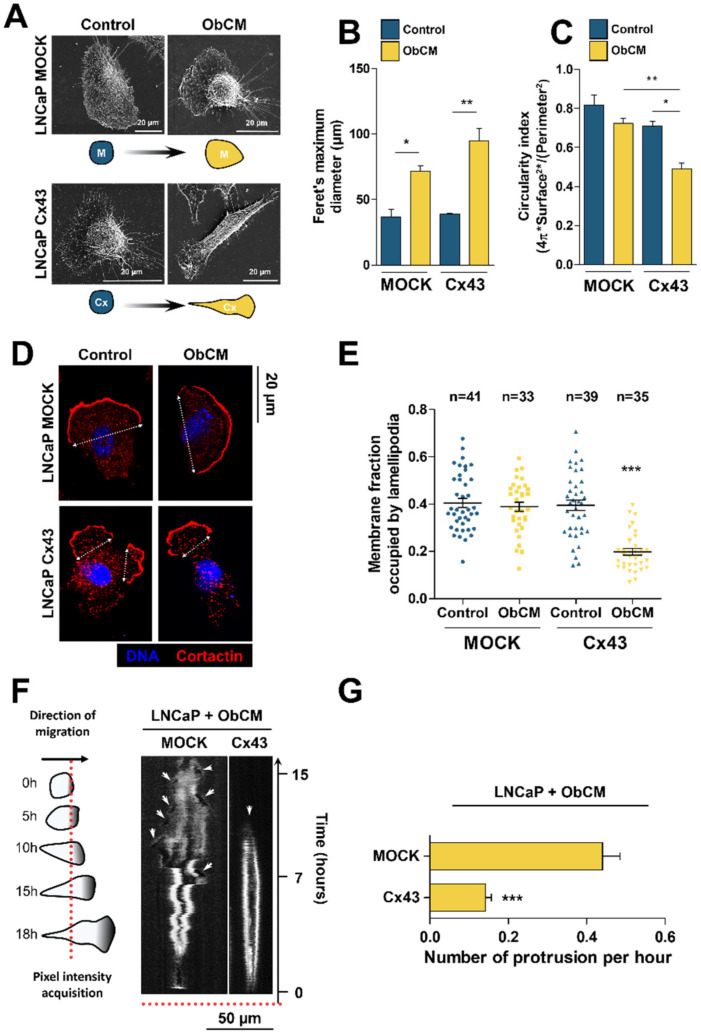
ObCM induces protrusive membrane dynamic in LNCaP Cx43 cells. Representative scanning electron microscopic images of LNCaP cells after 17 h of stimulation with or without ObCM (**A**) and associated quantification of cell morphology with Feret’s maximum diameter (**B**) and circularity index (**C**). Data represent the mean ± SEM from 3 different experiments. (**D**) Immunostaining of cortactin (red) highlighting lamellipodia in LNCaP cells after 17 h of stimulation with or without ObCM; nuclei were DAPI-stained (blue). (**E**) Quantification of the membrane fraction occupied by such membrane specialization. Data represent the mean ± SEM from indicated number of quantified cells. (**F**) Representative kymographs from LNCaP cells upon ObCM stimulation showing protrusion dynamics over time on a single image; red dotted line represents ROI used to perform the *x-t* scan necessary to generate such a graph. (**G**) Quantification of the mean number of protrusions emitted by one cell per hour. * p < 0.05; ** p < 0.01; *** p < 0.001.

To validate our results, we provide a quantitative analysis of LNCaP cells morphological changes associated with ObCM stimulation. Using light and electron microscopy, we found that, in control conditions, there was no cell shape difference between LNCaP overexpressing Cx43 and LNCaP control cells (Figure 4A and Figure S5). However, upon a 17 h exposure to ObCM, LNCaP Cx43 cells presented an elongated shape and acquired a mesenchymal-like phenotype more pronounced than LNCaP mock cells. The measurement of the circularity index and the Feret’s diameter confirmed these observations and statistical differences linked to Cx43 overexpression (Figure 4B,C).

Directional and efficient migration relies on polarized dynamic rearrangement of cortical actin cytoskeleton resulting in the formation and extension of cellular protrusions, such as lamellipodia and filopodia. We therefore examined the lamellipodia formation during ObCM-induced PCa cells migration by immunofluorescence staining of cortactin to determine its subcellular localization (Figure 4D). The focalization of cortactin immunostaining at the leading edge of migrating LNCaP Cx43 cells in the presence of ObCM is significant of polarized protrusive membrane formation (Figure 4E). Quantification of normalized leading edges length revealed that under ObCM stimulation, Cx43-overexpressing cells had more focalized lamellipodia than mock cells. To address the dynamics of these protrusions over time, we performed a kymograph-like analysis of the migration front of LNCaP cells under stimulated conditions with ObCM (Figure 4F,G). Consistent with cortactin localization profile, this analysis clearly showed linear extension of a single persistent protrusion in LNCaP Cx43 cells. Conversely, several multidirectional and unpersistent lamellar protrusions were typically observed in mock cells as illustrated by the number of protrusions emitted per hour determined with phase contrast microscopy.

Altogether, these data indicate that Cx43 can influence the single cell motility of prostate cancer cells in a bone microenvironment.

### 2.3. Membrane Localization of Cx43 Is Required for the Promigratory Effect of Bone-Conditioned Medium but Gap Junctional Intercellular Communication or Hemichannel Permeability Are Not Involved

**Figure 5:**
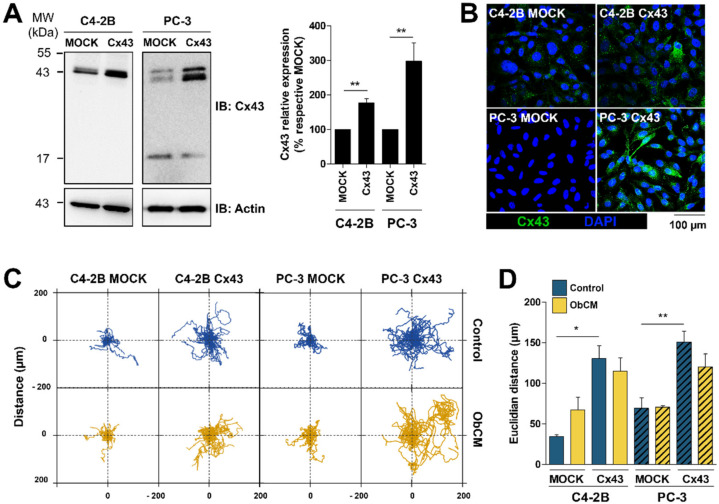
Impaired membrane targeting of Cx43 prevents osteoblasts-dependent migration in two other human prostate cancer (PCa) cell lines. (**A**) Representative immunoblots and densitometric quantification of Cx43 in PCa cells stably transfected to overexpress Cx43. Data represent the mean ± SEM from 7 (C4-2B) or 4 (PC-3) different experiments. (**B**) Immunostaining of Cx43 (green) in C4-2B and PC-3 MOCK and Cx43 cells (green); nuclei were DAPI-stained (blue). (**C**,**D**) quantitative analysis of single cell migration of C4-2B and PC-3 cells using time-lapse videomicroscopy. Cells were incubated for 17 h with either control media or osteoblasts-conditioned media (ObCM) and records were used for manual tracking of cell centroid trajectories. (**C**) Scaled spider graph showing for each condition and over 17 h of recording the individual paths of 15 representative cells, starting from their initial position at T = 0. (**D**) Euclidian distance (μm) has been measured and relative graph is shown. The values represent the mean ± SEM from 3 different experiments (C4-2B MOCK CTRL *n* = 49; ObCM-treated C4-2B MOCK *n* = 44; C4-2B Cx43 CTRL *n* = 79; ObCM-treated C4-2B Cx43 *n* = 91; PC-3 MOCK CTRL *n* = 78; ObCM-treated PC-3 MOCK *n* = 70; PC-3 Cx43 CTRL *n* = 98; ObCM-treated PC-3 Cx43 *n* = 119). * p < 0.05; ** p < 0.01.

Given that Cx43 could be present both at the plasma membrane and in the cytoplasmic part of PCa cells, we conducted a series of experiments to test the influence of Cx43 localization in the promigratory effect induced by ObCM. Therefore, two others human PCa cell lines (C4-2B and PC-3) were transfected to stably overexpress Cx43. The characterization showed an altered traffic of Cx43 to the plasma membrane (Figure 5A,B) that led to an absence (PC-3) or a half-reduction (C4-2b) of functional GJIC compared to the LNCaP model (Supplementary Figure S6). Videomicroscopy experiments aiming at measuring single cell migration of these two other PCa cell lines upon ObCM stimulation were performed. Cx43-overexpression induced a significant increase in migration capacities compared to mock cells (Figure 5C,D), as illustrated by spider graphs and associated quantifications (Euclidian distance improved by 1.8- and 3.8-fold for PC-3 and C4-2B, respectively). However, in contrast with the LNCaP cells, ObCM had no additional effect on the cellular dynamic of both PC3 and C4-2B cell lines, suggesting that membrane localization of Cx43 is required for sensitivity to bone microenvironment.

It is now well established that Cx43 can exert its functions at the plasma membrane through GJIC between cells, hemichannel activity and as a site for protein-protein interaction to regulate signaling pathways. In our study, we can rule out the first hypothesis that Cx43 is implicated in ObCM-induced migration of PCa cells by forming gap junctions since our migration experiments were performed with low cell density, which consequently prevented collective migration and direct intercellular exchange of molecules (see Figure S5).

**Figure 6:**
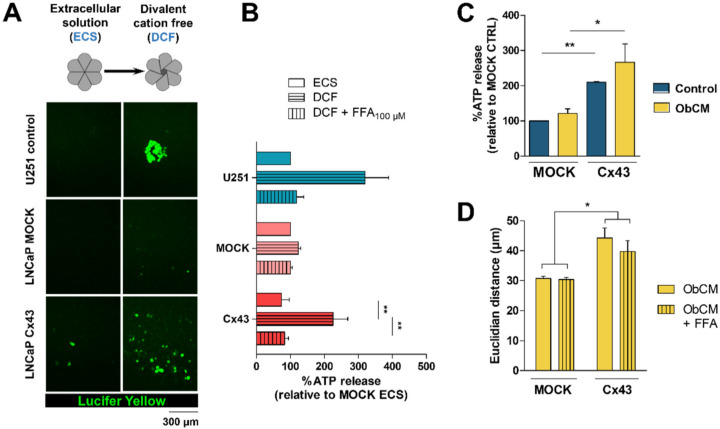
LNCaP Cx43 express functional hemichannels that do not contribute to ObCM induced migration. (**A**) Representative images of Lucifer Yellow incorporation assays performed with LNCaP cells. Cells were incubated in defined physiological solutions containing (ECS) or not divalent cations (DCF), allowing for the control of hemichannels opening. The hemichannel permeant dye tracer Lucifer Yellow (0.5 mg/mL) was added and uptaken by cells if hemichannel were functional. The glioblastoma cell line U251 expressing high levels of Cx43 and exhibiting high rates of Lucifer yellow (LY) uptake served as a positive control. (**B**) Biochemical assessment of hemichannel opening by means of ATP release quantification. The cells were treated as described in (**A**) with the addition of flufenamic acid (FFA 100 μM), a hemichannel blocker. Cell supernatants were collected after 15 min and extracellular concentrations of ATP were determined by a luciferin-luciferase based assay. For each condition, luminescence values were normalized by total protein amount. Data represent the mean ± SEM from 3 different experiments, except for U251 (*n* = 2). (**C**) Quantification of extracellular ATP after incubation of LNCaP cells with control media or ObCM. For each condition, luminescence values were normalized by total protein amount. Data represent the mean ± SEM from 3 different experiments. (**D**) quantitative analysis of single cell migration of LNCaP upon stimulation with ObCM with or without FFA (100 μM). Data represent the mean ± SEM from 3 different experiments (ObCM-treated LNCaP MOCK *n* = 69; ObCM + FFA-treated LNCaP MOCK *n* = 65; ObCM-treated LNCaP Cx43 *n* = 76; ObCM + FFA-treated LNCaP Cx43 *n* = 55). * p < 0.05; ** p < 0.01.

Then, to address the presence of functional hemichannels in LNCaP mock and LNCaP Cx43, we studied Lucifer yellow incorporation and the efflux of ATP in divalent cation free solution (DCF). The U251 human malignant glioblastoma cell line was used as control (Figure S7A) since Cx43 hemichannel activity has been previously well described in these cells [28]. As shown in Figure 6A, overexpression of Cx43 in LNCaP cells increased Lucifer Yellow uptake after 15 min of incubation in DCF. In accordance with hemichannel opening, Cx43 overexpression induced a significant increase of ATP release in LNCaP cells, which was totally prevented by a Cx/pannexin channel inhibitor, flufenamic acid (FFA) (Figure 6B).

Moreover, no difference in pannexin-1 expression, another hemichannel-forming protein, was demonstrated between LNCaP-mock and LNCaP-Cx43 cells suggesting that the hemichannel activity established in the former was effectively due to Cx43-hemichannels (Supplementary Figure S7B). We then tested whether Cx43-dependent migration induced by ObCM could rely on hemichannel activity. While Cx43 overexpression in LNCaP cells increased ATP release when incubated in control medium, no additional effect could be demonstrated after ObCM stimulation (Figure 6C). Consistently, in those stimulatory conditions, FFA had no significant inhibitory action on LNCaP Cx43 cells migration, as quantified by Euclidian distance (Figure 6D). These results suggest that exchanges between intra- and extracellular spaces via hemichannels are unlikely to contribute to this pro-migratory effect.

### 2.4. Carboxy-Terminal (CT) Domain of Cx43 Is Essential for the Promigratory Effect of Bone-Conditioned Medium

**Figure 7:**
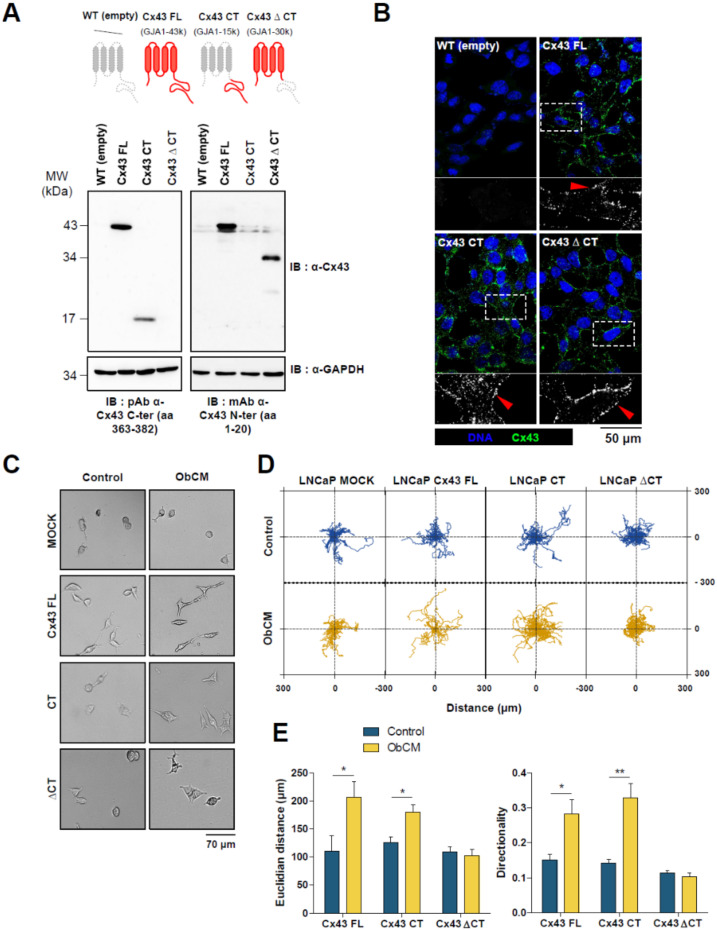
Channel mutants lacking carboxyl-tail of Cx43 fails to increase ObCM-induced migration. (**A**) Representative immunoblots of Cx43 in LNCaP cells transfected to stably express either full length human Cx43 (FL, 382 aa, 43kDa), C-ter truncated (CT, 139 aa, 15 kDa) or N-ter truncated (∆CT, 242aa, 30 kDa) versions of the protein; left: Immunoblot with anti-Cx43 C-ter antibody and right: Immunoblot with anti-Cx43 N-ter antibody. (**B**) Immunostaining of Cx43 and its truncated forms in LNCaP cells (green); nuclei were DAPI-stained (blue). Enlarged images marked by white box show higher magnification of Cx43 staining for each cell line: Red arrowheads showing that Cx43 immunoreactivity was typically present as puncta at the cell surface. (**C**) Brightfield images of LNCaP cells expressing truncated versions of Cx43 after 17 h of stimulation with or without ObCM. (**D**,**E**), Quantitative analysis of single cell migration of LNCaP cells using time-lapse videomicroscopy. Cells were incubated for 17 h with either control media or osteoblasts-conditioned media (ObCM) and records were used for manual tracking of cell centroid trajectories. (**D**) Scaled spider graph showing the individual paths of 15 representative cells, starting from their initial position at T = 0. (**E**) quantification of Euclidian distance and directionality. Data represent the mean ± SEM from 3 different experiments (Cx43 FL CTRL *n* = 143; Cx43 FL ObCM *n* = 99; CT CTRL *n* = 165; CT ObCM *n* = 161; ∆CT CTRL *n* = 97; ∆CT ObCM *n* = 131). * p < 0.05; ** p < 0.01.

Finally, we hypothesized that Cx43 affected the dynamic abilities of LNCaP cells in bone context by channel-independent functions linked to number of potential interaction sites of its CT fragment. We therefore stably transfected LNCaP cells with either full-length Cx43 (Cx43 FL) or two truncated Cx43 constructs (Figure 7A) corresponding to CT domain alone from amino acid 243 (Cx43 CT) or to channel-forming sequence truncated at amino acid 242 (Cx43 ∆CT). The parental cell line LNCaP transfected with the empty plasmid vector (LNCaP mock) expressing a low amount of endogenous Cx43 was used as control for characterization.

Western blot analyses using antibodies directed against either the N- or the C-terminal domain confirmed that the recombinant Cx43 proteins were overexpressed at expected sizes (43 kDa for Cx43 FL, 30 kDa for Cx43∆CT, and 15 kDa for Cx43 CT) (Figure 7A). Transfection of Cx43 ∆CT and Cx43 CT in LNCaP cells did not alter the level of endogenous Cx43 compared to mock cells.

We next examined localization of Cx43 by immunofluorescence (Figure 7B) and confirmed that LNCaP mock cells expressed very low amount of endogenous Cx43, localized both in the cytoplasm and at the plasma membrane. In LNCaP cells transfected with Cx43 FL, we observed a stronger signal localized predominantly in zones of cell-cell contacts. In contrast, the Cx43 CT truncated protein accumulated mostly in the cytoplasm but plasma membrane staining was also clearly evidenced. Finally, as for the full-length protein, the ∆CT mutant was predominantly found at plasma membrane. GJIC capacities of the different clones were compared to mock cells by means of preloading assay (Figure S8). As expected, a large increase in intercellular communication was demonstrated for Cx43 FL-transfected cells (+73.6% of coupled cells and +319% of calcein diffusion rate). In accordance with IF staining, Cx43 CT cells presented a reduced GJIC with −37% and −43%, respectively, for the same parameters. For Cx43 ∆CT cells, analyses showed a slight increase in the number of coupled cells (+22%) but a significant rise of GJIC ability (+189% of calcein diffusion area) linked to the formation of well-designed intercellular channels. Observations under phase contrast microscope also revealed morphological disparities between LNCaP cells transfected with different forms of Cx43. Indeed, after a 17 h stimulation with ObCM, Cx43 CT-transfected LNCaP cells were elongated exhibiting a mesenchymal-like phenotype already observed for LNCaP cells transfected with Cx43 FL (Figure 4A), whereas a more rounded shape similar to mock cells was shown for Cx43 ∆CT-transfected LNCaP cells (Figure 7C).

All these clones were then used to analyze the promigratory effect of ObCM by means of time-lapse videomicroscopy. As previously demonstrated above, the full-length form of Cx43 conferred to LNCaP cells sensitivity to ObCM (Figure 7D) leading to improved directionality and Euclidian distance (Figure 7E). Deletion of the carboxyl tail of the protein totally abolished the promigratory effect of the bone-conditioned medium while the CT portion alone was sufficient to maintain this phenotype with the same magnitude as for Cx43 FL-overexpressing cells.

Altogether, these results demonstrated that Cx43 CT is necessary for the responsiveness of PCa cells to ObCM.

### 2.5. Cx43 Interacts with Cortactin and Activates the Rac1/PAK1 Pathway under Bone-Conditioned Medium Stimulation

**Figure 8:**
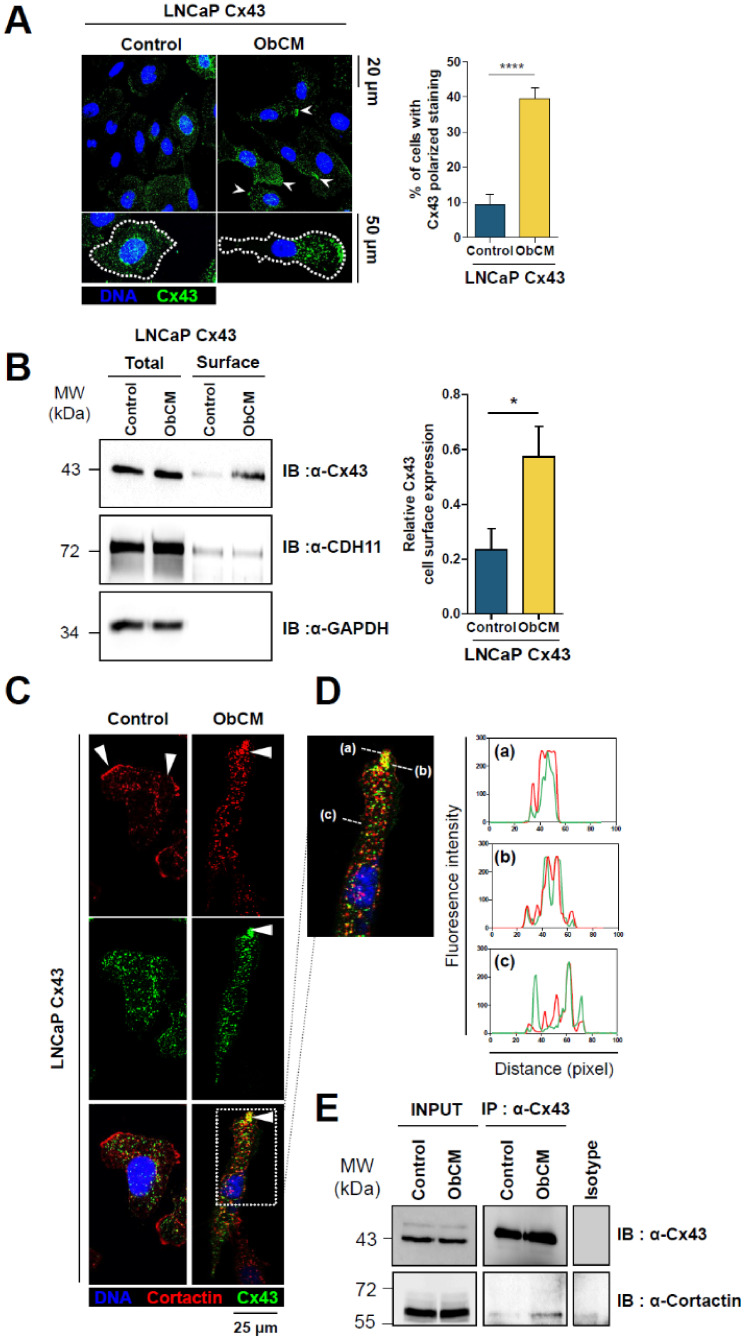
Cx43 enriched upon ObCM stimulation colocalizes in lamellipodia with Cortactin. (**A**) (Left) Immunostaining of Cx43 (green) in LNCaP Cx43 cells after 17 h of stimulation with or without ObCM; nuclei were DAPI-stained (blue). White arrowheads show areas of accumulated staining corresponding to the front of migration of cells, e.g., lamellipodia. Enlarged images show higher magnification of Cx43 staining in the 2 conditions. (Right) Quantification of the percentage of cells with Cx43 staining being polarized to the leading edge of the cells (CTRL, *n* = 89; ObCM, *n* = 169). (**B**) Representative immunoblot (left) and densitometric quantification (right) after surface biotinylation assay showing an increase in Cx43 enrichment at the surface of LNCaP Cx43 cells after 17 h of incubation with ObCM. Pre- (total) and post-Streptavidin pull down protein lysates (Surface) from LNCaP Cx43 CTRL and LNCaP Cx43 ObCM were immunoblotted with indicated antibodies. Cadherin 11 served as a positive control of cell surface expression. Data represent the mean ± SEM from 3 different experiments. (**C**) Co-immunostaining of Cx43 (green) and cortactin (red) in LNCaP Cx43 cells after 17 h of stimulation with or without ObCM; nuclei were DAPI-stained (blue). (**D**) Enlarged image shows higher magnification of Cx43 and cortactin staining in LNCaP Cx43 cells stimulated with ObCM. Fluorescence intensity profiles along the segments (**a**–**c**) suggest a colocalization of Cx43 with Cortactin at the leading edge of the cells. (**E**) Interaction between Cx43 and cortactin in LNCaP Cx43 was assessed by immunoprecipitation 17 h after stimulation of cells with or without ObCM. Pre- (total) and post-immunoprecipitation protein cell extracts were immunoblotted with indicated antibodies. Isotype refers to immunoprecipitation control with irrelevant antibodies. * p < 0.05; **** p < 0.0001.

As for well-characterized regulators of the cytoskeletal reorganization machinery, Cx43 subcellular localization might be critical to perform its dedicated function in ObCM-induced migration. Therefore, we thought to determine whether the pro-migratory effect could be associated with any changes in Cx43 subcellular location. Our immunofluorescence analysis performed in LNCaP Cx43 cells revealed an asymmetric distribution of Cx43 staining along the front-rear axis upon ObCM stimulation, with apparent greater concentration of Cx43 at the leading edge as quantified by the percentage of cells with polarized staining (Figure 8A). This focalization of Cx43 was associated with a specific enrichment at the cell surface (3-fold increase) without apparent change in the total expression level, as revealed by cell surface biotinylation experiments (Figure 8B). This particular localization of Cx43 at the leading edge of LNCaP cells suggests that it might be important for the control of migration.

Since a lamellipodia induction by ObCM was previously demonstrated, we investigated the possible interaction between Cx43 and cortactin during stimulation of PCa cells migration. A restricted colocalization of Cx43 and cortactin in protrusive membrane (Figure 8C,D) and an interaction by means of co-immunoprecipitation (co-IP) (Figure 8E) were demonstrated in LNCaP Cx43 stimulated with ObCM, supporting a role for Cx43 in the control of formation of lamellipodia and modulation of migration in a bone context.

**Figure 9:**
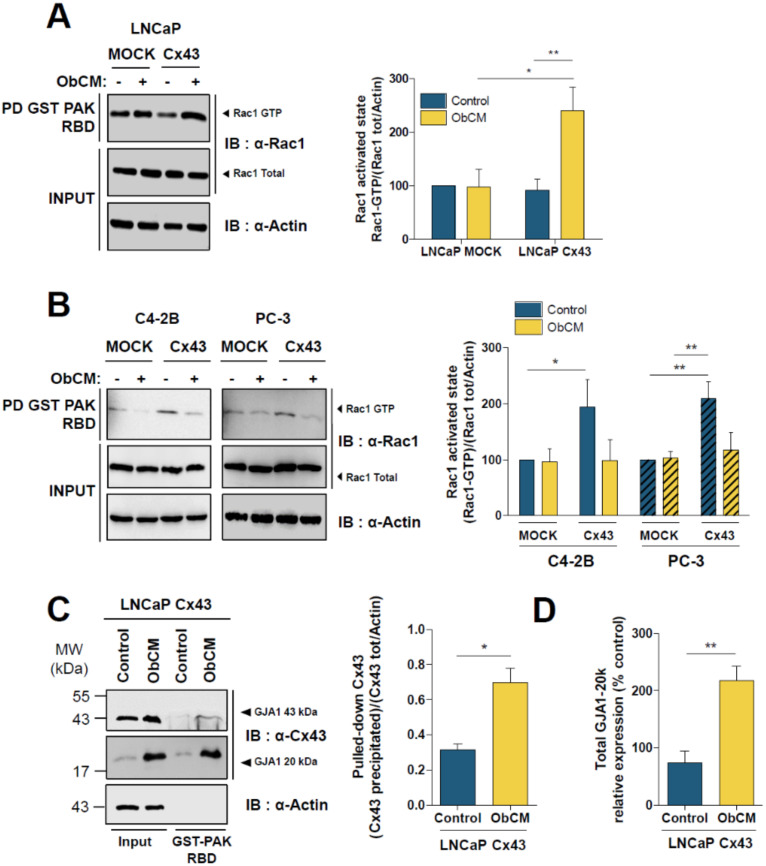
ObCM-induced migration of Cx43 targeting plasma membrane relies on Rac1 pathway. (**A**) Representative immunoblot and densitometric quantification of Rac1 (total and activated pool) as determined by GST pull down in LNCaP cells after 17 h of stimulation with or without ObCM. Cell lysates were incubated with sepharose-bound GST-fused PAK-RBD and either total cell lysate (input) or pulled-down bead-bound complexes comprising activated form of Rac1 (GTP-bound, Pulldown GST-PAK-RBD) were immunoblotted with anti-Rac1 antibody. Data represent the mean ± SEM from 4 different experiments. (**B**) Rac1 activated state was assessed as described in (**A**) in PC-3 and C4-2B overexpressing Cx43. Data represent the mean ± SEM from 4 different experiments. (**C**) representative immunoblot and densitometric quantification of Cx43 (total and PAK-RBD-interacting pool) as determined by GST pull down in LNCaP cells after stimulation with or without ObCM. Cells were treated as in (**A**), but immunoblotting was done with anti-Cx43 antibody. Actin served as control, showing no binding to GST-PAK-RBD. (**D**) densitometric quantification of GJA1-20k in LNCaP Cx43 after ObCM stimulation. Data represent the mean ± SEM from 4 different experiments. * p < 0.05; ** p < 0.01.

As Rac1 is known to induce cortactin localization to the cell membrane and mediate lamellipodia formation, we examined in LNCaP mock and LNCaP Cx43 cells the level of active Rac1 level in basal condition and after ObCM stimulation by means of pull-down assay. As shown in Figure 9A, the activation level of Rac1 was similar between unstimulated LNCaP mock and LNCaP Cx43 cells. However, after ObCM treatment, this level significantly increased by 2.6-fold only in LNCaP Cx43 cells.

With the PCa cell lines presenting an altered Cx43 traffic to the plasma membrane and insensitive to ObCM in terms of migration (PC-3 and C4-2b), Cx43 overexpression led to an increase of the Rac1 activated state (Figure 9B) which was inhibited after ObCM treatment. These GST-PAK-RBD pulldown assays also revealed that Cx43 was a partner of Rac-1, which is pulldowned at higher level upon ObCM stimulation (Figure 9C). Interestingly, we also demonstrated that a Gja1-20K isoform was also highly precipitated by GST-PAK-RBD in LNCaP Cx43 cells. This isoform, known to be involved in full length Cx43 trafficking to the plasma membrane in other cell types [29], also exhibited an increased expression level in ObCM-stimulated LNCaP Cx43 cells.

## 3. Discussion

The mechanisms underlying Cx43 action in the progression of prostate cancer cells towards bones are incompletely deciphered. An ambiguous and spatiotemporal role for Cx43 during initiation and progression of solid cancers and dissemination of carcinoma cells is now well documented [6,7], with antitumoral features during the first steps mainly linked to antiproliferative effects, whereas later stages are rather associated with protumoral actions. To answer some of the outstanding questions about the role of Cx43 during the development of bone metastases, we first performed immunohistochemical assays on patient samples and we then analyzed the impact of Cx43-overexpression in different prostatic cell lines on their phenotypic features when placed in bone microenvironment reproduced by osteoblastic-conditioned medium.

As observed in most solid tumors and in accordance with previous observations in primary localized prostate tumors [11], our data showed a loss of Cx43 expression in PCa cells from patients during early stages compared to normal tissue or benign prostatic hypertrophy. The reasons leading to this Cx43 downregulation are not fully deciphered. Androgens, which are linked to the development of prostate cancer [29], may act by repressing Cx43 expression through androgen receptor pathway [30]. More generally, GJA1 gene expression could be reduced at both transcriptional and posttranscriptional levels involving transcriptional repressors, promoter methylation and mRNA degradation by miRNA [31]. We also revealed a Cx43 re-expression in advanced histological grades (notably grade 4), whereas, in a retrospective and a single protein analysis, a decreased expression level was associated with high Gleason score and worse prognosis by Xu et al. (2016) [23]. The main contribution of our retrospective analysis of published databases was to unveil a specific Cx43 signature in bone metastasis compared to other metastatic sites at the transcript and protein levels. These results confirm the assumption that Cx43 is implicated in pathophysiological processes linked to dissemination of PCa cells toward bones [16]. Such a Cx43 expression profile has already been demonstrated in bone metastatic breast cancer cells [32], but in this context Cx43 was considered as an osteoblastic differentiation marker reflecting an osteomimetic phenotype for cancer cells more than an active factor of metastatic acquisition. The Cx43 expression profiles in various PCa cell lines reinforce the hypothesis of an important role of Cx43 in the bone metastatic context as only bone-targeting cell lines (C4-2B and PC-3) exhibit an increased relative expression level compared to normal epithelial cells. Under normal growth culture conditions, Cx43 expression level in the LNCaP model was very low, as recently published [14] and shown in the present study. In a progression model based on cell lines, Tate et al. (2006) [33] also demonstrated that PC-3 metastatic cells (PC-3M) expressed higher levels of Cx43 transcripts than parental PC-3 cells.

Using Cx43-overexpressing low aggressive LNCaP cells, we confirmed that Cx43 expression level had no significant effects on LNCaP cells proliferation and apoptosis as previously shown by our group and others [14,15]. Interestingly, no influence on these cellular functions could be demonstrated under osteoblast-conditioned medium (ObCM) treatment. Regarding the invasion process, we revealed a stimulating action of ObCM similar whatever Cx43 expression level, while Cx43 seems to be required for invasive phenotype and for invadopodia formation in other cancer types, such as breast and gliomas [28,34,35,36]. Accumulating evidences have revealed an emerging role of Cx43 in promoting cell migration in normal development [37], as well as during progression of different types of cancer, including glioma, melanoma, and breast cancer (for review: Reference [38]. For prostate cancer, we previously demonstrated a correlation between Cx43 expression levels with the metastatic potential of LNCaP cells [15]. Moreover, Zhang et al. [14] positively correlated the malignancy potential of PCa cells with Cx43 expression level among the 7 Cxs expressed in prostate tissues. By a sh-RNA approach on PC-3 cells, these authors confirmed the implication of Cx43 in the migration ability of PCa cells.

Nonetheless, the hypotheses of authors working in field of cancer differ regarding the mechanisms employed by Cx43 to potentiate cell migration processes. Indeed, GJIC, hemichannel functionality or intracellular signaling linked to CT-Cx43 have all been suggested to be critical in promoting cell motility. Since the observed effects were obtained using single cells, GJIC cannot account for the Cx43 promigratory action. In our study, no hemichannel activity (ATP and purinergic receptors) could be implicated in the promigratory action as demonstrated by pharmacological treatment using flufenamic acid. Moreover, in the context of direct communication between PCa and osteoblastic cells during bone metastasis development, GJIC was mainly implicated in the dormancy or reactivation of cancer cells [39]. Interestingly, osteogenic cells can promote bone metastasis from breast or prostate origin by direct calcium influx through gap junctions into cancer cells [22]. Both in physiological and pathological situations, it becomes now clear that Cx43 expression at the plasma membrane level mainly influences the forward cell movement and the directionality of cellular protrusions by intracellular activities [37]. As in other models (breast cancer and lymphoma [38]), our data obtained with PCa cells support a role for Cx43 in the regulation of the actin cytoskeleton network, in the stabilization of cell protrusions and intrinsic directed migration. Abnormal actin organization and reduced directionality have also been observed in neural crest cells derived from Cx43 knockout mice [40]. Therefore, it is conceivable that Cx43 modulates cell motility by affecting directly or indirectly the cytoskeletal network. A dynamic remodeling of the actin network occurs during cancer cell migration and invasion [41], during which the small GTPases signaling cascades are the main regulators of actin reorganization [42]. Typically, actin filament polymerization induced by either the Cdc42 or Rac-mediated cascades results in the emergence of filopodia and lamellipodia at the leading edges [43]. As in other cell types, a clear correlation exists between migration abilities and activated Rac-1 level in PCa cells. In PC-3 cells, the overexpression of constitutively active Rac1 resulted in significant increase in cell migration as recently demonstrated [44], and, in the present study, Cx43 overexpression in PC-3 or C4-2B cells was able to enhance concomitantly Rac-1 and migration activities. In the LNCaP model, we also demonstrated a direct link between Cx43 and active Rac-1 together with a decreased dynamic of lamellipodia. Moreover, Cx43 that colocalized with cortactin in protrusive membrane of LNCaP cells optimized the directionality under ObCM stimulation. However, other intracellular proteins implicated in metastatic progression and able to interact with Cx43 (tubulins, ezrin, ZO-1, vimentin, vinculin; for review, see Reference [16]) cannot be excluded and their roles should be tested.

It is now well documented that the C-tail of Cx43 is the domain that interact directly with various cytoskeleton proteins implicated in cell motility (reviewed in Reference [45,46,47]). Using truncated forms of Cx43, we demonstrated that the CT domain (aa 243–382) is essential and sufficient for the promigratory effects and corroborated the Cx43 CT domain implication in migration previously demonstrated in Hela cells [20,48]. The enhanced migration of cells expressing either full length Cx43 or Cx43 CT was associated with an increased activation of the p38 MAP kinase in HeLa cells [48]. Interestingly, we observed the same result with LNCaP cells in preliminary experiments [49]. Therefore, the unique functions of Cx43 CT on migration process could be carried out both as an essential component of full-length Cx43 and as an independent signaling hub. Recently, a direct regulation of N-cadherin transcription after translocation of Cx43 carboxy tail to the nucleus was demonstrated to impact neural crest cell migration in vivo [50]. Moreover, high proportion of prolines and serines has important physiological consequences and makes this part of Cx43 a potential target for extensive post-translational modifications that modulate its intracellular trafficking [9]. Interestingly, after transfection of the human GJA1 gene in PCa cells, we revealed the presence of a Cx43 isoform at 20 kDa (Gja1-20k) expressed at high level in LNCaP cells, at lower level in PC-3 cells and absent in C4-2b cells. This internally translated Cx43 short isoform was implicated in the full-length Cx43 anterograde traffic to the plasma membrane [51,52], in its delivery to cardiac intercalated discs [53], and in its protection from degradation [54]. Such a control of Cx43 membrane localization by Gja1-20k was also demonstrated during TGFβ-induced epithelial to mesenchymal transition of mammary cells [55]. As PC-3 and C4-2b cells present exportation defects of the full-length form of Cx43, our results argue for a potential role played by Gja1-20k and confirm that post-transcriptional mechanisms based on chaperon action or on microtubules stabilization exist to limit the presence of Cx43-FL at the plasma membrane. Moreover, under ObCM stimulation, the promigratory effect in LNCaP cells correlates with an increased expression level of Gja1-20k together with an increase of membranous Cx43. To decipher further the role of Gja1-20k in the proactive effect of Cx43 on directional migration, we plan to overexpress Gja1-20k in traffic-deficient cell lines (PC-3 or C4-2b) and to analyze the impact on Cx43 localization and ObCM sensitivity.

To our knowledge, the present study represents the first demonstration of a role for Cx43 in sensitivity of prostate cancer cell migration to osteoblastic-conditioned medium. Different osteoblastic-secreted factors were already demonstrated to affect PCa cells proliferation and apoptosis processes [56,57], dissemination towards bones [58,59], their adherence [60], or their survival/dormancy [61] in bone context. Among them, bone-specific like osteonectin/SPARC (secreted protein, acidic and rich in cysteine) and nonspecific factors like TGF-beta1 present in ObCM were shown to increase migration of PCa cells and to induce formation of a mesenchymal-like phenotype [62,63]. Collectively, these findings indicate that osteoblastic cells produce factors that are able to modify the migration capability of prostate cancer cells and our data suggest that Cx43 may be an important element of the metastatic risk by modulating sensitivity to these bone factors. ObCM sensitivity requires the membrane localization for Cx43 given the absence of promigratory effects of ObCM in PC-3 and C4-2b cells. As a perspective, we obviously plan to identify the main osteoblastic factors involved in the Cx43-dependent promigratory effect by proteomic analyses of ObCM. Among the potential bone factors, Wnt5a and CCL5 were recently implicated in the promotion of PCa cells migration [64,65]. In preliminary data, we pointed out periostin or Osteoblast Specific Factor 2 (OSF-2) that presents an expression level correlated with Gleason score and poor prognosis [66]. This chemo-attractive molecule, produced by reactive stroma of other aggressive carcinomas, was implicated in the control of cell migratory abilities [67,68]. This factor, also present in ObCM produced by preosteoblastic cells in our culture conditions, is able to increase expression level and localization of Cx43 at plasma membrane in migratory front of LNCaP Cx43 cells and to induce alone a rise in directional migration [49]. Extracellular vesicles potentially present in ObCM could also be consider as modulators of cancer microenvironment [69]. Crosstalk between PCa cells and mesenchymal stem cells via exosomes containing miRNAs have already been demonstrated [70]. Finally, the differential sensitivity demonstrated between LNCaP cells could be due to a specific surfaceome profile induced by the presence of increased level of Cx43 at the plasma membrane and could be investigated using an RNA-seq and cell surface proteomic approach.

## 4. Materials and Methods

### 4.1. Antibodies

Mouse monoclonal antibodies directed against Cx43 N-ter (#MABT903, clone P1E11.B19), mouse monoclonal anti-Cortactin (#05-180) and mouse monoclonal antibody anti-Rac1 (#23A8) were purchased from Merck-Millipore (Darmstadt, Germany). Rabbit polyclonal antibody directed against Cx43 C-ter (#C6219) and mouse monoclonal antibody anti-Actin ((#A5441) were supplied by Sigma (St. Louis, MO, USA). Mouse monoclonal antibody anti-β1Integrin was from Abcam (#ab30394, Cambridge, England). Mouse monoclonal antibody directed against Cx43 C-ter was purchased from BD Transduction Laboratories (#610062, San Jose, CA, USA). Mouse monoclonal against Glyceraldehyde-3-phosphate dehydrogenase (GAPDH) was from Hytest (#5G4, Turku, Finland).

### 4.2. Plasmid Constructs

Plasmids with pMSCV-puro backbone (Clonetech laboratories, Palo Alto, CA, USA, containing cDNA for full-length (FL, aa 1-382) or for the two truncated versions (CT, aa 243–382 and ∆CT, aa 1–382) of human Cx43 were a kind gift of Dr Wun-Chey Sin (Vancouver, BC, Canada) and characterized in Reference [43].

### 4.3. Cell Culture

LNCaP, PC-3, DU-145, and RWPE-1 cell lines were purchased from American Type Culture Collection (ATCC, Manassas, VI, USA; CRL-1740, CRL-1435, HTB-81, and CRL-11609 respectively) and C4-2B cell line was kindly provided by Dr. Potier-Cartereau (Tours, France). All cells were cultured at 37°C in a 5% CO_2_ incubator. LNCaP, PC-3, and DU-145 cells were maintained in Dulbecco’s modified Eagle’s medium (DMEM; Thermo Fisher Scientific, Waltham, MA, USA) containing 200 mM GlutaMAX, 0.1 g/L sodium pyruvate, 4.5 g/L D-glucose and supplemented with 10% Fetal Bovine Serum (FBS), 100 IU/mL penicillin, and 100 μg/mL Streptomycin. C4-2B cells were grown in Roswell Park Memorial Institute medium RPMI-1640 (Thermo Fisher Scientific, Waltham, MA, USA) containing 200 mM GlutaMAX, 2 g/L D-glucose and supplemented as described above. RWPE-1 cells were maintained in Keratinocyte Serum Free Medium (K-SFM) supplemented with 30 μg/mL Bovine Pituitary Extract (BPE), 0.2 ng/mL Epidermal Growth Factor (EGF), and 5 μg/mL Gentamycin.

LNCaP and PC-3 cells stably expressing human Cx43 (LNCaP Cx43 and PC-3 Cx43) were previously established by means of retroviral particles and described in Reference [15]. C4-2B cells expressing human Cx43 were obtained by transfecting parental cell lines with empty or Cx43 cDNA-containing pMSCV-puro using FuGENE HD transfection reagent according to the manufacturer’s instructions (Promega, Madison, WI, USA). Transfected cells were selected for 2 weeks with 5 μg/mL puromycin (Sigma). LNCaP cells expressing Cx43 FL, CT, and ΔCT forms of human Cx43 were also obtained by transfection as described above.

Murine primary osteoblastic cells (OB) were isolated as previously described [15]. Briefly, OB cells were isolated from calvaria of 3–5 days-old mice after sequential digestions with trypsin and collagenase. After grown to confluency at 37 °C in phenol-red free DMEM containing 200 mM GlutaMAX, 4.5 g/L D-glucose and supplemented with 20% FBS, 100 IU/mL penicillin, and 100 μg/mL streptomycin, OB cells were then seeded and maintained for 5 days in differentiating medium (phenol-red free DMEM supplemented with 10% FBS and 10 mM sodium β-glycerophosphate (Sigma). The media was change once, after day 2 of differentiation. Conditioned medium was then collected, centrifuged sequentially at 300 and 14,000 *g* and then kept at −80 °C. This medium was referred to ObCM in this study.

### 4.4. Immunohistochemistry on Patient Samples

Investigations were carried out following the rules of the Declaration of Helsinki. Human samples were obtained from a tissue collection of patients treated at the university hospital of Poitiers (CHU La Milétrie, Poitiers, France) and permission to use this materiel was obtained from the ethical board of the Centre de Ressources Biologiques (CRB, Poitiers, France; ethical code number: BB-0033-00068, date of approval: 9 June 2017). Signed informed consent of patients were obtained by the local Ethic Committee and our request for additional data was approved under Project “Cx43 and bone metastasis” by the CNIL (Commission Nationale de l’Informatique et des Libertés, Paris, France, date of approval: 27 April 2017). To avoid pharmacological bias, patients included in the study never underwent hormonotherapy, chemotherapy or antiresorptive treatments. Blocks from surgical excision were cut 5-μm thick and stained with hematoxylin-eosin. Five-milliliter tissue sections were placed on charged slides, baked at 60 °C overnight, then deparaffinized and rehydrated. After blocking with Bovine Serum Albumin (BSA; Sigma, St. Louis, MO, USA) solutions and permeabilization in 0.5% Triton X-100 diluted in PBS, samples were incubated overnight using the mouse monoclonal antibody directed against Cx43 C-ter (Transduction Laboratories, San Jose, CA, USA) diluted at 1:200. Staining procedure used the ChemMate Detection kit (DakoCytomation, Glostrup, Denmark), based on an indirect biotin-avidin system with a universal biotinylated immunoglobulin secondary antibody, diaminobenzidin (DAB) substrate, and hematoxylin counterstain. Original slides from the 35 samples were reviewed by a pathologist to locate the tumor area before microphotographs acquisition.

### 4.5. Western Blot

Cells were lysed in ice cold RIPA buffer containing 50 mM Tris HCl pH7.4, 15 mM NaCl, 5 mM EDTA, 0.05% NP-40, 0.5% Sodium deoxycholate, 1% Triton X100, 0.01% SDS, and completed by 1 x protease and phosphatase inhibitors cocktail (Roche Applied Science, Penzberg, Germany). Protein extracts were cleared from cell debris by centrifugation at 12,500× *g* for 10 min at 4 °C. Protein concentration was measured using DC protein assay kit (Bio-Rad Laboratories, Hercules, CA, USA). Equal amount of proteins from different samples were mixed with 5X Laemmli-loading buffer (150 mM Tris-HCl, pH 6.8, 5% SDS, 12.5% 2-mercaptoethanol, 25% glycerol, and 0.025% bromophenol blue) to get a 1× final concentration.

Proteins were resolved using 10% SDS-PAGE gels and transferred to PVDF membranes (Merck Millipore, Darmstadt, Germany). Membranes were blocked for 2 h at room temperature (RT) with 5% (*w*/*v*) non-fat powdered milk in Tris-buffered saline-Tween (TBS-T; 25 mM Tris-HCl, pH 8.0, 15 mM NaCl, 0.01% Tween 20) and incubated overnight at 4°C with primary antibodies diluted in TBS-T containing 5% (*w*/*v*) non-fat milk. The primary antibody concentration was as follows: 0.25 μg/mL monoclonal anti-Cx43 CT, 1:50 dilution for monoclonal anti-Cx43 NT, 1 μg/mL dilution for anti-IntegrinB1, 0.5 μg/mL anti-cadherin11, 1:1000 dilution for anti-cortactin, anti-Cx43 CT, anti-Rac1, anti-GAPDH, and anti-Actin. Membranes were then washed with TBS-T and then incubated for 1 h at RT with corresponding secondary horseradish peroxidase-conjugated anti-mouse or anti-rabbit antibodies (1:10,000) for 1 h. Immunodetection was performed using chemiluminescent Luminata Forte substrate (Merck Millipore, Darmstadt, Germany) and LAS-3000 imaging system (Fujifilm, Tokyo, Japan). Densitometric analysis of signals was carried out using ImageJ software (version 1.39, National Institutes of Health, Bethesda, MD, USA).

### 4.6. Immunocytofluorescence

After grown at low density on glass coverslips, LNCaP cells were treated with or without ObCM for 17 h before fixation in ice-cold 1:1 mix of methanol/acetone for 10 min. Cells were then blocked with 1% BSA, 1% Triton X100 in PBS at room temperature for 1 h. LNCaP cells were incubated overnight at 4 °C with mouse monoclonal anti-Cterm Cx43 or anti-Nterm Cx43, and mouse monoclonal anti-Cortactin antibodies prepared in blocking buffer at 1:250 dilution. A negative control was performed by omitting primary antibodies. LNCaP cells were then incubated with either Alexa Fluor 555 or 488 anti-Mouse secondary antibodies (1:1000) in blocking buffer for 2 h at RT. Coverslips were mounted with vectashield- Di-Aminido-Phenyl-lndol (DAPI) fluorescent mounting medium (Vector Laboratories, Burlingame, CA, USA) allowing nuclei staining. Images were captured using a confocal microscope (Olympus FV1000, Tokyo, Japan).

### 4.7. Cell Surface Biotinylation

To determine cell surface expression of Cx43, 0.8 × 10^6^ LNCaP cells were seeded in 6 cm-diameter culture dishes. After adhesion to plastic, cells were stimulated under the same conditions as for migration. After 17 h of stimulation with or without ObCM, cells were rinsed twice at 4 °C with biotinylation buffer containing 0.1 mM CaCl_2_, 1 mM MgCl_2_, PBS pH7,4. Cells were then incubated with EZ-LINK sulfo-NHS-SS-Biotin (Thermo Fisher Scientific, Waltham, MA, USA) for 1 h at 4 °C with gentle rocking. The labelling reaction was quenched for 10 min at 4 °C with biotinylation buffer containing 0.1% BSA. Cells were then washed twice in biotinylation buffer and were lysed in RIPA buffer. Protein concentration of each sample was determined by DC Protein Assay kit at 750 nm. Forty micrograms of the cell lysate was kept for total quantification of Cx43, Integrin-B1, Cadherin-11, and GAPDH expression by immunoblot. In parallel, 100 μg of proteins from the cell lysate were incubated together with 5% (*v*/*v*) Streptavidin-Agarose beads in RIPA buffer at 4 °C overnight with gentle rocking. Biotin-Streptavidin complexes were then washed twice with RIPA buffer and biotinylated were eluted with 2× Laemmli-sample buffer. Total lysates and biotinylated proteins were analyzed by Western blot with anti-Cx43, Integrin-B1, Cadherin-11, and GAPDH, as described in Section 4.5.

### 4.8. Integral Membrane Protein Extraction

Integral membrane proteins and membrane-associated proteins from LNCaP cells were extracted by using Mem-PER Plus membrane protein extraction kit (Thermo Fisher Scientific, Waltham, MA, USA), according to the manufacturer’s instructions. One hundred micrograms of membrane-protein extracts and 40 μg of proteins from whole cell lysates were prepared in Laemmli sample buffer and resolved by 12% SDS-PAGE prior to immunoblotting with anti-Cx43 and anti-Cadherin11, as previously described in Section 4.5.

### 4.9. Single Cell Migration by Time-Lapse Videomicroscopy

For single cell migration assessment, tumor cell lines were seeded in osteoblastic differentiation medium in 6-well plates at low density (1500 cells/cm^2^) and were allowed to adhere on plastic. After 7 h, cell proliferation capacities were inhibited by a treatment with a non-cytotoxic concentration (10 μg/mL) of mitomycin-C for 1 h. Cells were then stimulated with either fresh OB differentiation medium or ObCM. Time-lapse recording was performed with JuLI^TM^Stage device (NanoEnTek, Seoul, Korea) in optimal cell culture conditions (37 °C, 5% CO_2_, humidified atmosphere). Images were acquired on 4 random fields in each well with a 10x brightfield objective, at a frame rate of 1 at 3-min intervals for 17 h. Image analysis was performed by manual tracking of cell centroid for all time points of the recording using ‘Manual Tracking’ plugin from ImageJ. All cells of a given field were analyzed excluding those which paths were interrupted during the recording. X; Y coordinates files obtained were then imported with the recommended format into ‘Chemotaxis and Migration Tool’ software (version 2.0, Ibidi GmbH, Martinstried, Germany). Calculated dynamic parameters, such as Euclidian distance, accumulated distance, and directness, were extracted after calibration of the software with appropriate settings (pixel size and time interval). Data represent the mean values of dynamic parameters from 3 independent experiments. After 15 h of recording, three morphological parameters were also quantified for all cells in a given field with ImageJ software: circularity index as defined by 4 × π × area/perimeter (AU), Feret’s maximum diameter (μm), and cell perimeter (μm). Data represent the mean values of morphological parameters from 3 independent experiments.

### 4.10. Scanning Electron Microscopy (SEM)

LNCaP cells were fixed for 1 h with 2.5% glutaraldehyde in 0.1 M phosphate buffer, pH 7.1. After PBS washes, dehydration was carried out using successive incubations of increasing ethanol concentrations (from 50% to 100%). Cells were dried by hexamethyldisilazane treatment and sputter-coated in a vacuum with an electrically conductive 3-nm thick layer of platinum. SEM images were then recorded with a scanning electron microscope, TENEO Volumescope of ThemoFisher scientific at 5 KV voltage.

### 4.11. Lucifer Yellow Uptake

To evidence the presence of functional hemichannels in Cx43 overexpressing LNCaP cells, we experimentally induced hemichannels opening by using calcium-free extracellular solutions. For this, 4 × 10^4^ LNCaP or U251 cells (serving as a positive control of functional hemichannels) in 1 mL growth medium were seeded in 24-well plates. Twenty-four hours later, cells were rinsed twice with regular extracellular solution (ECS; 142 mM NaCl, 5.4 mM KCl, 1.5 mM MgCl_2_, 2 mM CaCl_2_, 10 mM HEPES, 25 mM D-glucose, pH 7.35) [71]. Cells were then incubated at 37 °C for 15 min with either ECS (unstimulated condition) or divalent cation-free solution (DCF, which is ECS without MgCl_2_ and CaCl_2_ and with 2 mM EGTA; stimulated condition) supplemented with 0.5 mg/mL fluorescent dye Lucifer Yellow (Sigma). This step allowed the forced opening of the Cx43 hemichannels and, consequently, the entry of the fluorescent tracer into the cells. Cells were then washed twice in ECS before low magnification image acquisition using Olympus MVX10 macroscope (objective 1×/1; 22°C; medium: PBS; Camera: Hamamatsu ORCA-03G; cellSens Dimension Version 1.4 software Olympus, Tokyo, Japan).

### 4.12. ATP Release

For ATP release experiments, the protocol used was the same as for Lucifer Yellow uptake assays, except Lucifer Yellow was omitted. During the stimulation phase, cells were incubated with DCF solution together with a hemichannel inhibitor, Flufenamic Acid (FFA (Sigma)) used at 0.5 mg/mL. After 2 washes in ECS, extracellular medium was collected on ice and sequentially centrifuged at 300 and 14,000× *g* to remove cells and cellular debris, respectively. Extracellular ATP was quantified by ‘ATP determination kit’ according to the manufacturer’s instruction. This bioluminescence assay is based on ATP-dependent conversion of D-luciferin into oxyluciferin by luciferase leading to light emission. Luminescence was measured with luminometer microplate reader set at 560 nm. For each experimental condition, the amount of ATP (picomole) was calculated with a standard curve obtained from measurements of samples with known and increasing ATP concentrations. Those values were normalized by respective total protein amount determined by typical colorimetric assay (DC protein assay, Bio-Rad Laboratories) after cell lysis.

### 4.13. Immunoprecipitation Assay

To test interaction between Cx43 and cortactin, LNCaP cells stimulated with or without ObCM for 17 h were lysed with RIPA buffer. After protein concentration determination, 50 μg of the cell lysate were kept for total quantification of Cx43 and cortactin expression by immunoblot. In parallel, 500 μg of proteins were incubated 15 min at 4 °C with 1 μg of polyclonal rabbit anti-Cterm Cx43 or with irrelevant polyclonal rabbit antibody prepared in ice-cold RIPA buffer. Those samples were then incubated together with 50 μL of protein A/G-sepharose beads for 2 h at 4°C with gentle rocking to capture immune complexes. After washes in RIPA buffer, proteins were eluted from the beads by addition of 2× Laemmli loading buffer. The immunoprecipitates and proteins from whole cell lysates were analyzed by Western blot as described above.

### 4.14. GTP-Bound Rac1 Pull-Down Assay

The Glutathione S-transferase (GST)-tagged Rac-binding domain (RBD) of p21-activated kinase (PAK) [GST-PAK-RBD domain] was obtained as pGEX-2 T fusion genes (gift of JG Collard, Netherlands Cancer Institute, Amsterdam, NL, USA) and produced as described. Recombinant proteins were prepared as glutathione S-transferase fusion proteins in Escherichia coli (BL21 strain), purified using glutathione-sepharose beads (Amersham Pharmacia Biotech, Little Chalfont, UK), and used as GST-fusion proteins. To measure Rac1 activity, LNCaP cells were cultured and stimulated under the same conditions as for migration, except that they were seeded in 10 cm-diameter culture dishes. After 17 h of stimulation with or without ObCM, cells were lysed using a cell lysis buffer containing 50 mM Tris-HCl pH 7.4, 100 mM NaCl, 2 mM MgCl_2_, 1% NP-40 10% glycerol, and antiproteases cocktail. Protein concentration of each sample was determined by DC Protein Assay kit (Bio-Rad laboratories, Hercules, CA, USA) at 750 nm. Twenty micrograms of the cell lysate were kept for total quantification of Rac1 and actin expression by immunoblot. In parallel, 300 μg of proteins from the cell lysate were mixed with 30 μL of GST-PAK-RBD fusion protein coupled with glutathione-sepharose beads at 4 °C overnight with gentle rocking. Complexes containing GTP-bound Rac1 were then washed thrice with lysis buffer and proteins eluted with 2× Laemmli loading buffer. The pull-down complexes and proteins from whole cell lysates were fractionated by a 13% SDS–PAGE, followed by Western blotting with anti-Rac1 and anti-actin as described above. This approach was also used to detect Cx43 pulled-down with GST-PAK-RBD using Cx43 antibody instead of Rac1 antibody, as described in Reference [72].

### 4.15. Statistical Analyses

Results were expressed as the mean of n experiments ± the standard error of the mean (SEM). Statistical analyzes were carried out using GraphPad Prism software (version 5.0, GraphPad Software, San Diego, CA, USA) using data from at least 3 independent experiments. Analysis of variance (ANOVA, ANalysis of VAriance) followed by a Bonferroni post-test was used for the analysis of at least three experimental groups. Unpaired *t*-test was used to compare two experimental groups. Data were taken statistically significant for a value of *p* < 0.05 (* *p* < 0.05; ** *p* < 0.01; *** *p* < 0.001).

## 5. Conclusions

In the bone metastatic context, our results clearly demonstrate that Cx43 level and its membrane localization can modulate the phenotypic response of PCa cells to an osteoblastic microenvironment and that the CT domain of Cx43 plays a pivotal role in this phenomenon. Our study also reveals the presence of a N-terminally truncated Cx43 isoform (Gja1-20k) in LNCaP cells whose expression level depends on ObCM stimulation and may regulate the traffic of full-length Cx43 to plasma membrane and, subsequently, the promigratory effect.

## Supplementary Materials

The following are available online at https://www.mdpi.com/2072-6694/12/10/3013/s1, Figure S1: Complement to ‘GJA1 gene and protein expressions are associated with prostate cancer aggressiveness and bone metastases.’ Figure S2: Demonstration of homocellular GJIC ability in LNCaP Cx43 cells. Figure S3: Bone microenvironment has no effect on LNCaP cells viability and apoptosis regardless of Cx43 expression level. Figure S4: Osteoblasts increase LNCaP cells invasiveness regardless of Cx43 expression level. Figure S5: Complement to ‘ObCM induces protrusive membrane dynamic in LNCaP Cx43 cells. Figure S6: Lack of Cx43 cell surface expression and associated GJIC deficiency in PC-3 and C4-2B cell systems. Figure S7: Complement to ‘LNCaP Cx43 express functional hemichannels that do not contribute to ObCM induced migration.’ Figure S8: Evaluation of GJIC capacities in LNCaP cells expressing truncated versions of Cx43. Figure S9: Uncropped immunoblots.
