# Supplementary material for: Cx43 Present at the Leading Edge Membrane Governs Promigratory Effects of Osteoblast-Conditioned Medium on Human Prostate Cancer Cells in the Context of Bone Metastasis
Source: Cancers (Basel). 2020 Oct 16;12(10):3013. doi: 10.3390/cancers12103013 (PMC7602984; doi:10.3390/cancers12103013)

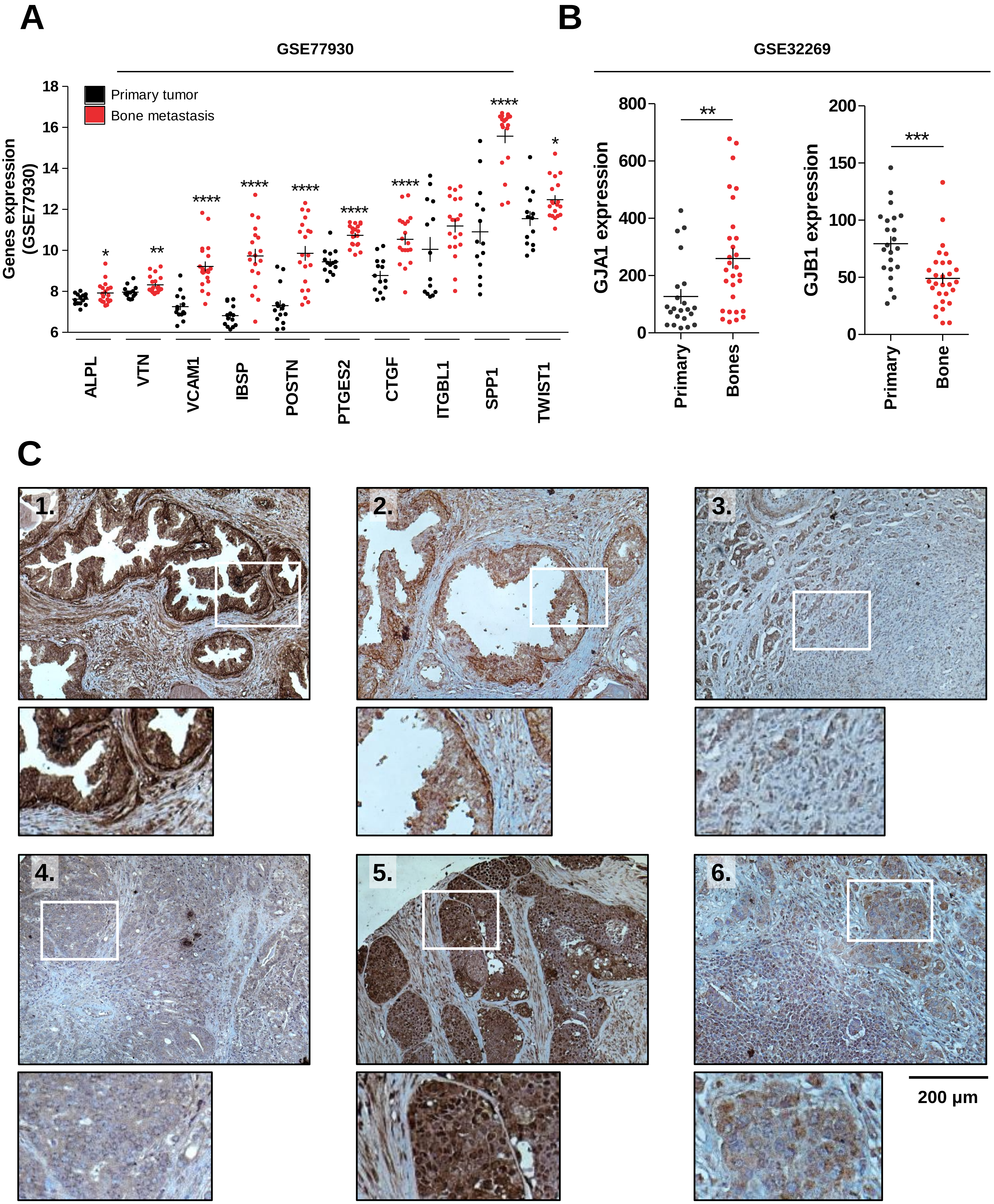

Figure S1: Complement to 'GJA1 gene and protein expressions are associated with prostate cancer aggressiveness and bone metastases.'

(A), GSE77930 gene expression analysis of bone versus primary samples showing increased expression of genes previously associated with bone metastasis of either prostate and breast carcinomas. **(B)**, GJA1 (left) and GJB1 (right) expression between primary localized prostate cancer (n=22) versus castration-resistant bone metastatic prostate cancer (n=29) (from GSE32269 dataset); ALPL, Alkaline Phosphatase; VTN, Vitronectin; VCAM1, Vascular Cell Adhesion Molecule 1; IBSP, Bone Sialoprotein II; POSTN, Periostin; PTGES2, Prostaglandin E Synthase 2; CTGF, Connective tissue growth factor; ITGBL1, Integrin Subunit Beta Like 1; SPP1, Secreted Phosphoprotein 1; TWIST1, Twist Family BHLH Transcription Factor 1. **(C)**, Cx43 expression by immunohistochemistry in TMA samples of prostate cancer: 1. Adjacent normal tissue; 2. Hyperplasia; 3. Grade IIa; 4. Grade III; 5. Grade IV; 6. Lymph Node.

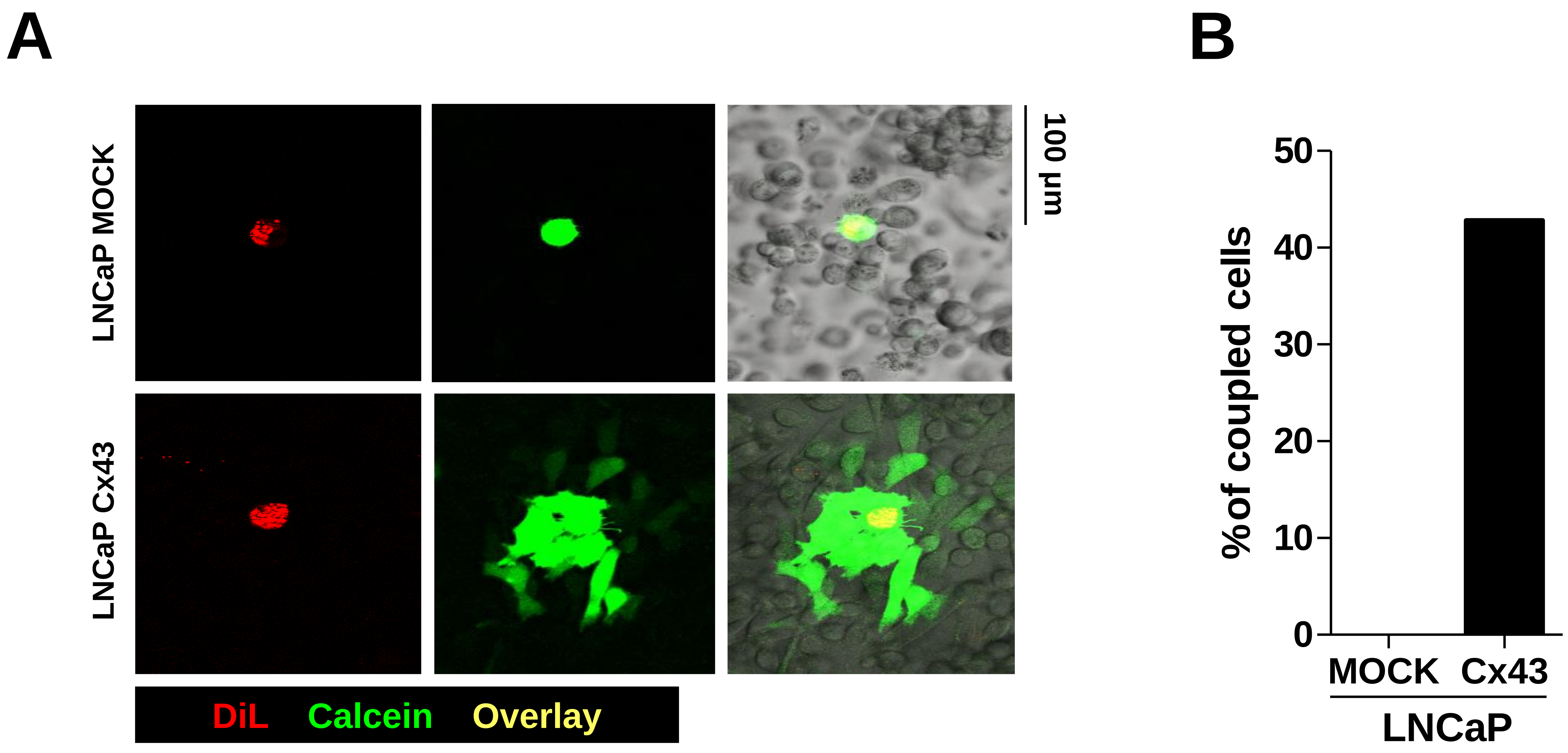

Figure S2: Demonstration of homocellular GJIC ability in LNCaP Cx43 cells.
(A), Demonstration of homocellular coupling between Cx43 overexpressing LNCaP cells by preloading assay. Only LNCaP Cx43 established GJIC with themselves as shown by calcein diffusion (green) from donor cells tagged by DiL-C18 (red). **(B)**, quantification plots showing the mean percentage of cells coupled.

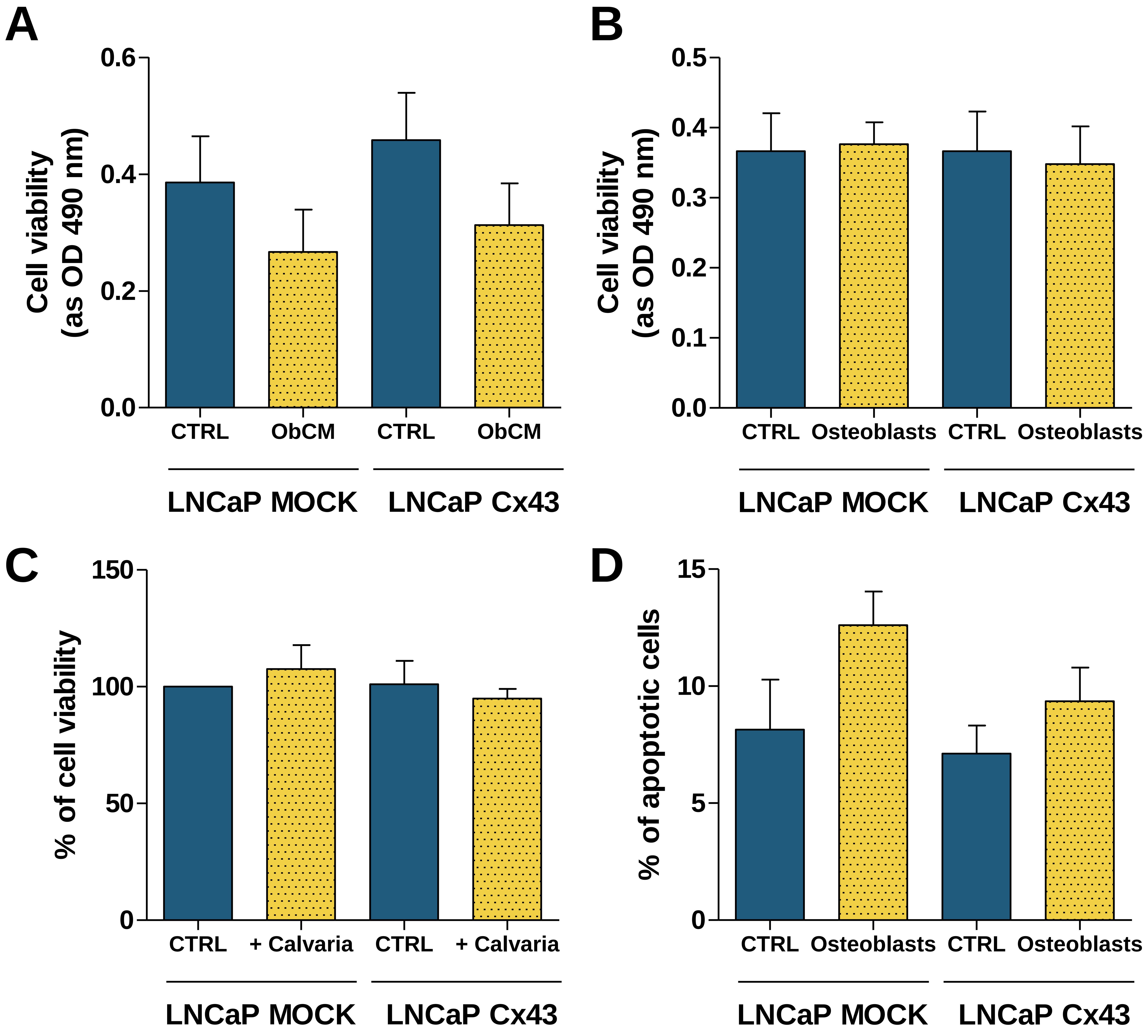

Figure S3: Bone microenvironment has no effect on LNCaP cells viability and apoptosis regardless of Cx43 expression level.

LNCaP cells viability was measured by XTT assay 48 hours after stimulation **(A)** with or without ObCM (N=5), **(B)** with or without differentiating osteoblasts (in coculture; N=3) and **(C)** with or without calvaria explants in coculture (N=3). **(D)**, LNCaP cell apoptosis was assessed 17 hours after coculture with or without osteoblasts by flow cytometry as a measurement of annexinV-positive cells (N=4). Data represent the mean ± SEM from indicated number of experiments.

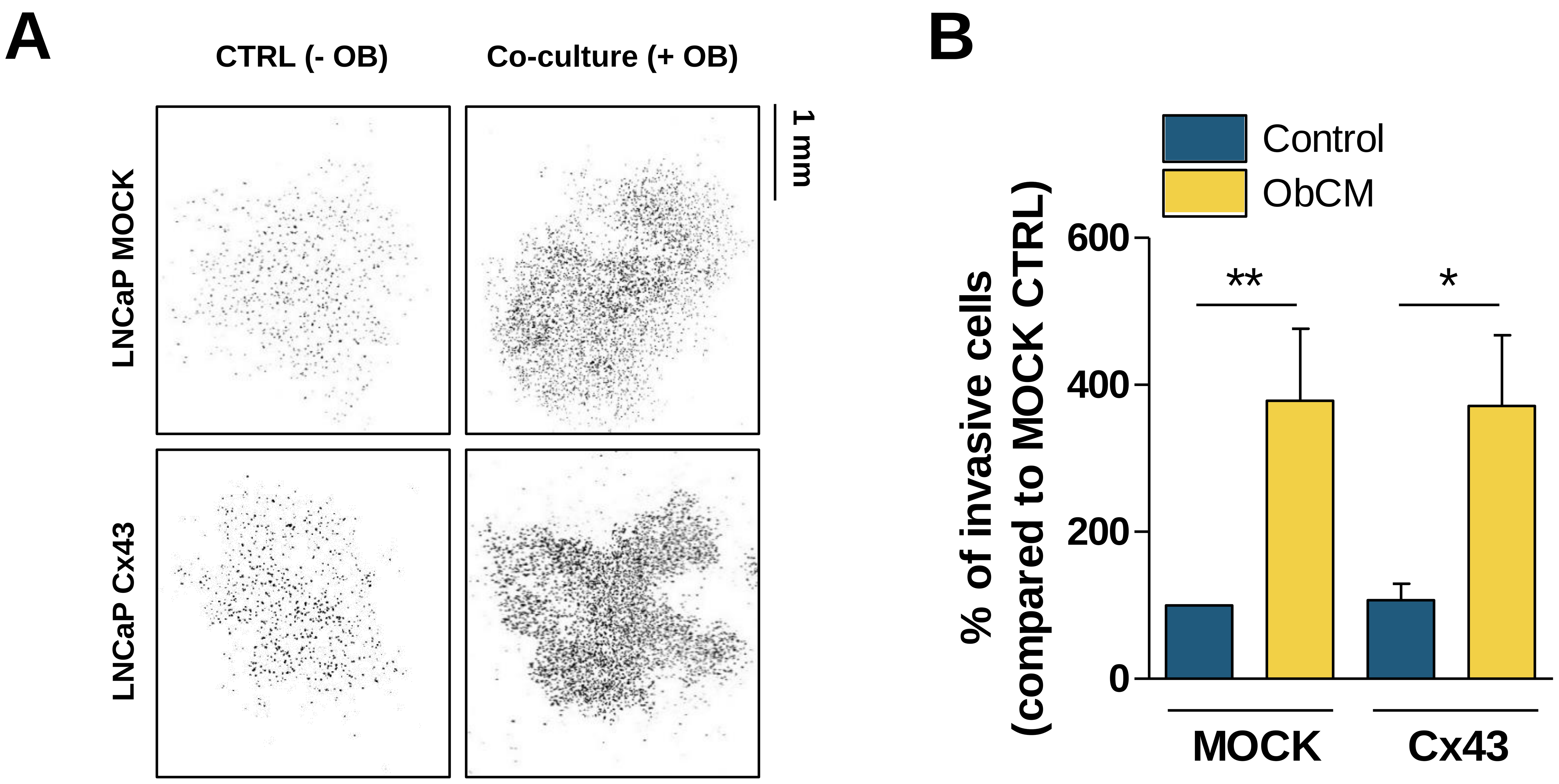

Figure S4: Osteoblasts increase LNCaP cells invasiveness regardless of Cx43 expression level.
LNCaP cells invasion through Matrigel® was measured after 17 hours of coculture using Boyden chambers with or without osteoblasts. **(A)** Representative images of DAPI-stained nuclei of invasive LNCaP cells that migrated through the membrane pores and **(B)**, quantification of the mean number of invading cells. Data represent the mean ± SEM from 8 different experiments. **p*<0.05; ***p*<0.01.

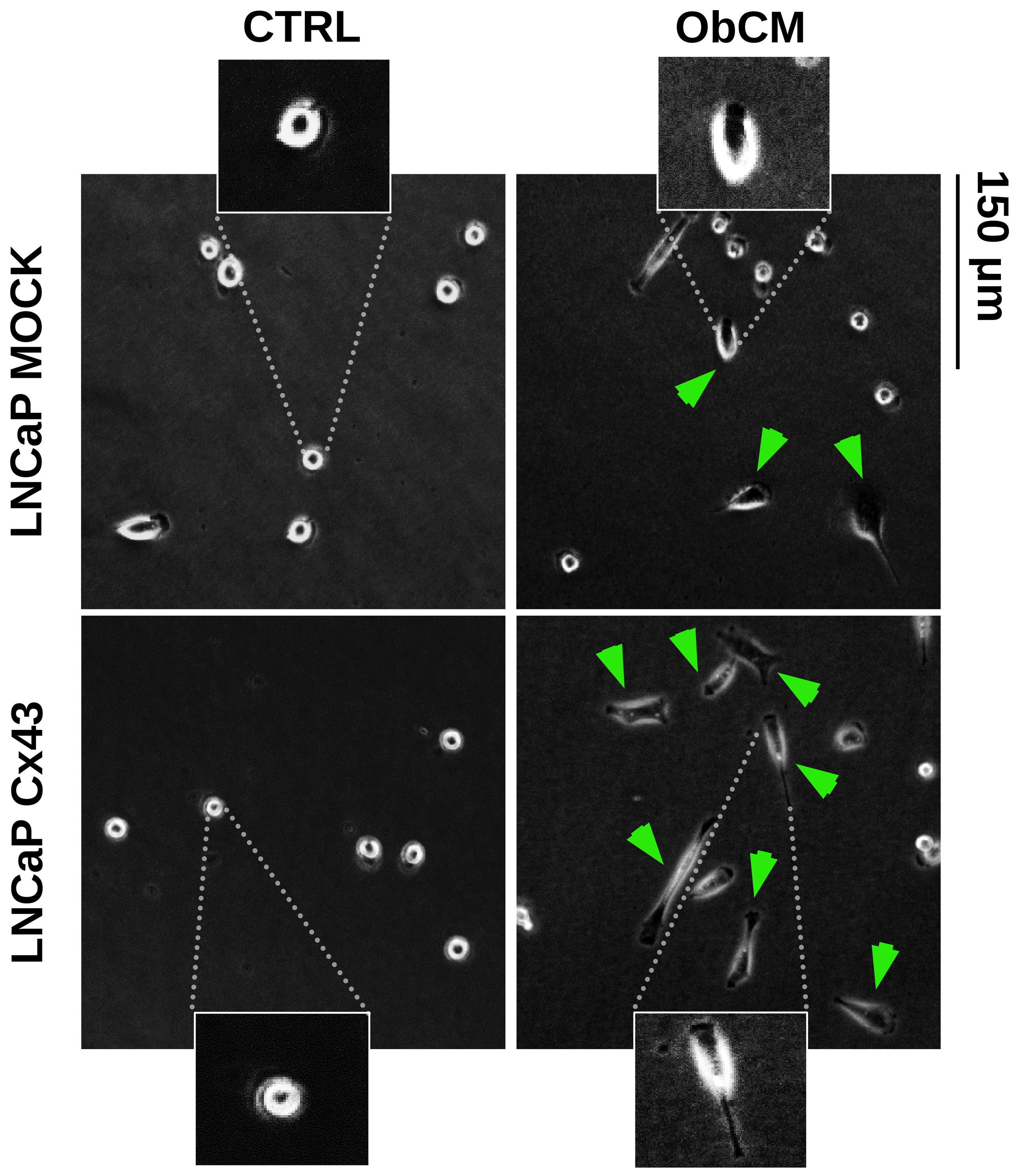

Figure S5: Complement to 'ObCM induces protrusive membrane dynamic in LNCaP Cx43 cells'. Phase contrast micrographs of LNCaP cells after 17 hours of stimulation with or without ObCM. Images show morphological differences described in figure 4 but at a larger scale. Green arrows show migrating cells with elongated cell body. LNCaP Cx43 cells exhibit a drastic elongation of cell body upon ObCM stimulation while LNCaP MOCK cell remain largely rounded.

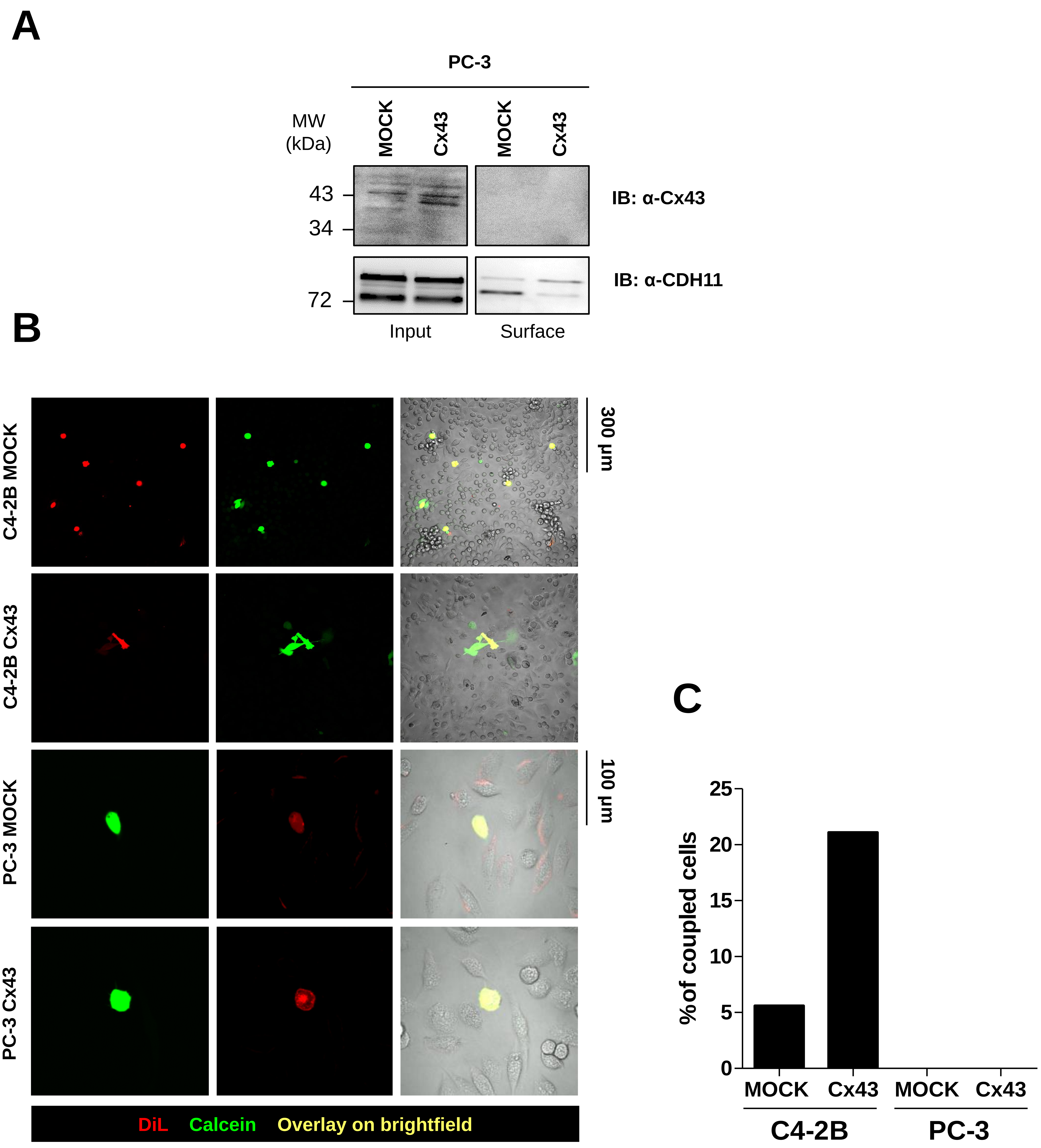

Figure S6: Lack of Cx43 cell surface expression and associated GJIC deficiency in PC-3 and C4-2B cell systems. **(A)**, Surface biotinylation assay and associated immunoblot showing an absence of Cx43 enrichment at the surface of PC-3 Cx43 cells. Pre- (total) and post Streptavidin pull down protein lysates (Surface) from PC-3 MOCK and Cx43 were immunoblotted with indicated antibodies. Cadherin 11 served as a positive control of cell surface expression. **(B)** and **(C)**, Homocellular coupling in C4-2B and PC-3 cells was assessed by preloading assays. **(B)**, representative images of calcein diffusion (green) in an acceptor monolayer of cells after 6 hours of co-culture with donor cells stained with both calcein (diffuse through gap junctions) and DiL-C18 (non diffusible lipophilic membrane marker). **(C)**, Quantification of the mean percentage of coupled cells. PC-3 cells did not show any obvious GJIC; A calcein diffusion occurring in 20% of cells could be evidenced in C4-2B Cx43, but still below than that for LNCaP Cx43.

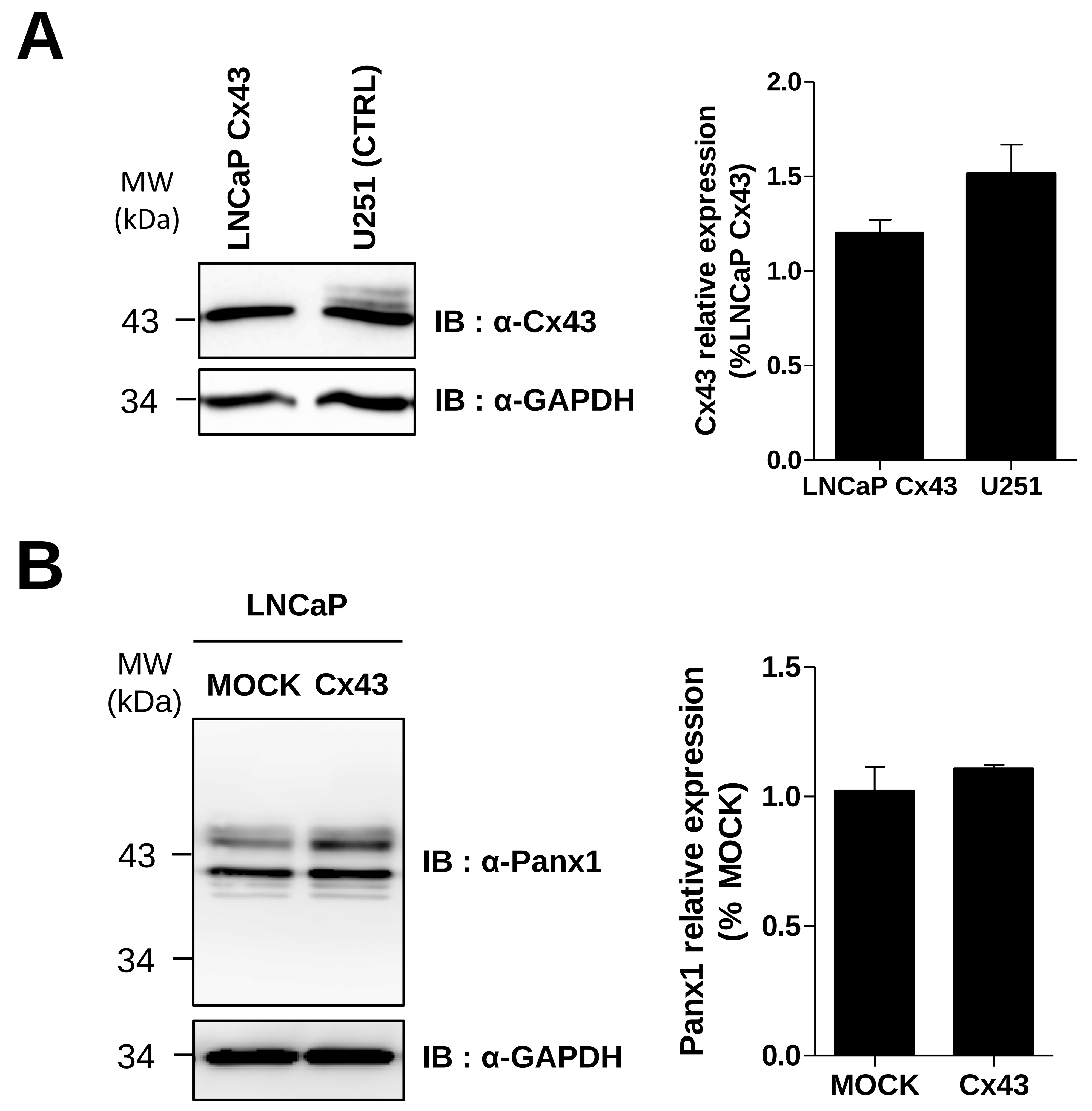

Figure S7: Complement to 'LNCaP Cx43 express functional hemichannels that do not contribute to ObCM induced migration.'

(A), Representative Western blot showing equal expression of Cx43 between LNCaP Cx43 and U251 cells. **(B)**, No modification of the pannexin-1 expression level after Cx43 overexpression in LNCaP cells illustrated by representative Western blot.

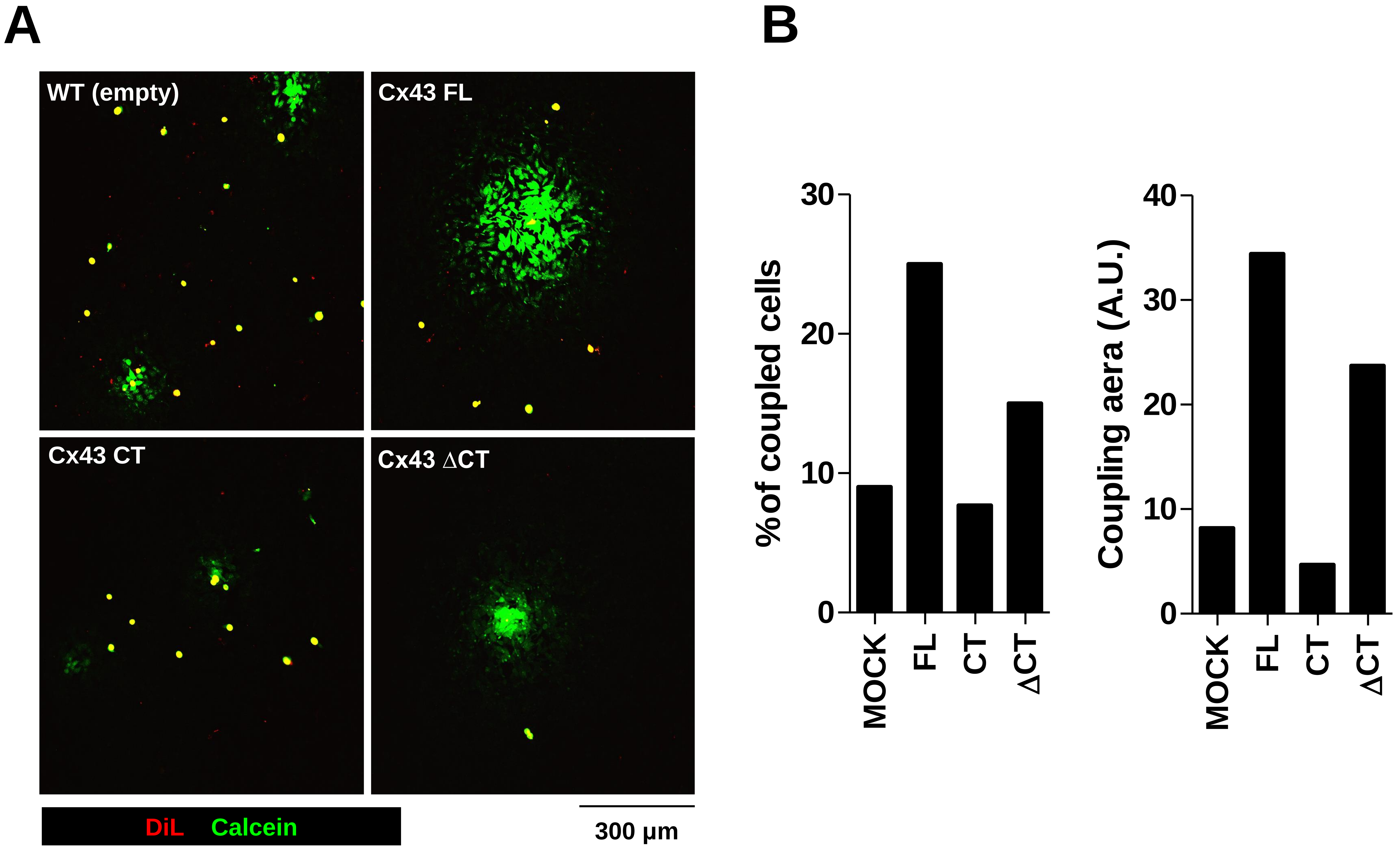

Figure S8: Evaluation of GJIC capacities in LNCaP cells expressing truncated versions of Cx43. Homocellular coupling in LNCaP cells expressing truncated variants of Cx43 was determined by preloading assays. (A), representative images of calcein diffusion (green) in an acceptor monolayer of LNCaP Cx43 cells after 6 hours of co-culture with donor cells stained with both calcein (diffuse through gap junctions) and DiL-C18 (non diffusible lipophilic membrane marker). (B), quantification of the mean percentage of coupled cells (number of diffusing parachuted cells/total number of parachuted cells) and the mean coupling area (fluorescent area originated by one parachuted cell). LNCaP MOCK and CT cells showed very limited GJIC capacities compared to LNCaP FL and to a lesser extent LNCaP ΔCT.

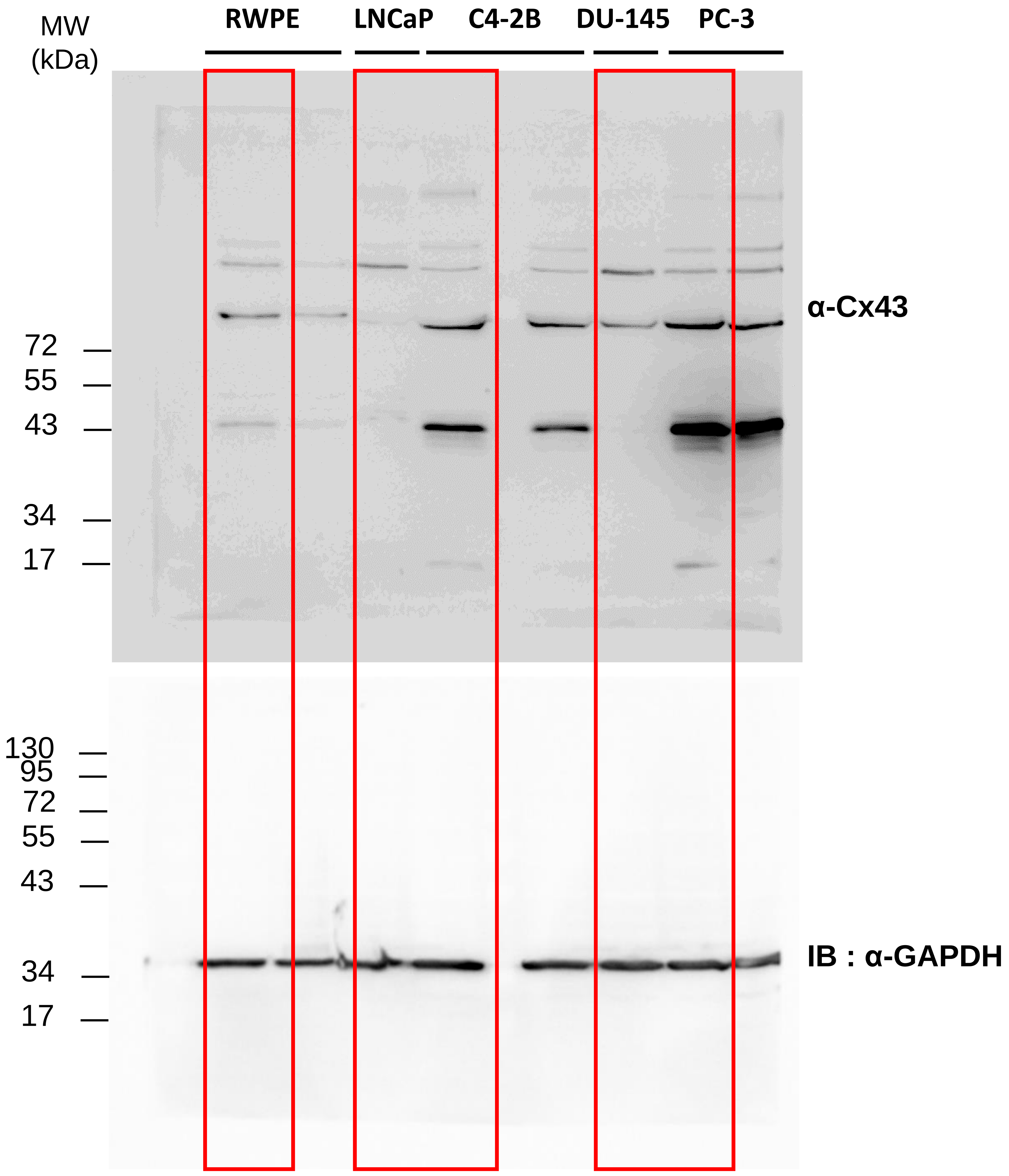

Uncropped immunblot from Figure 1C

Uncropped from Figure 2A

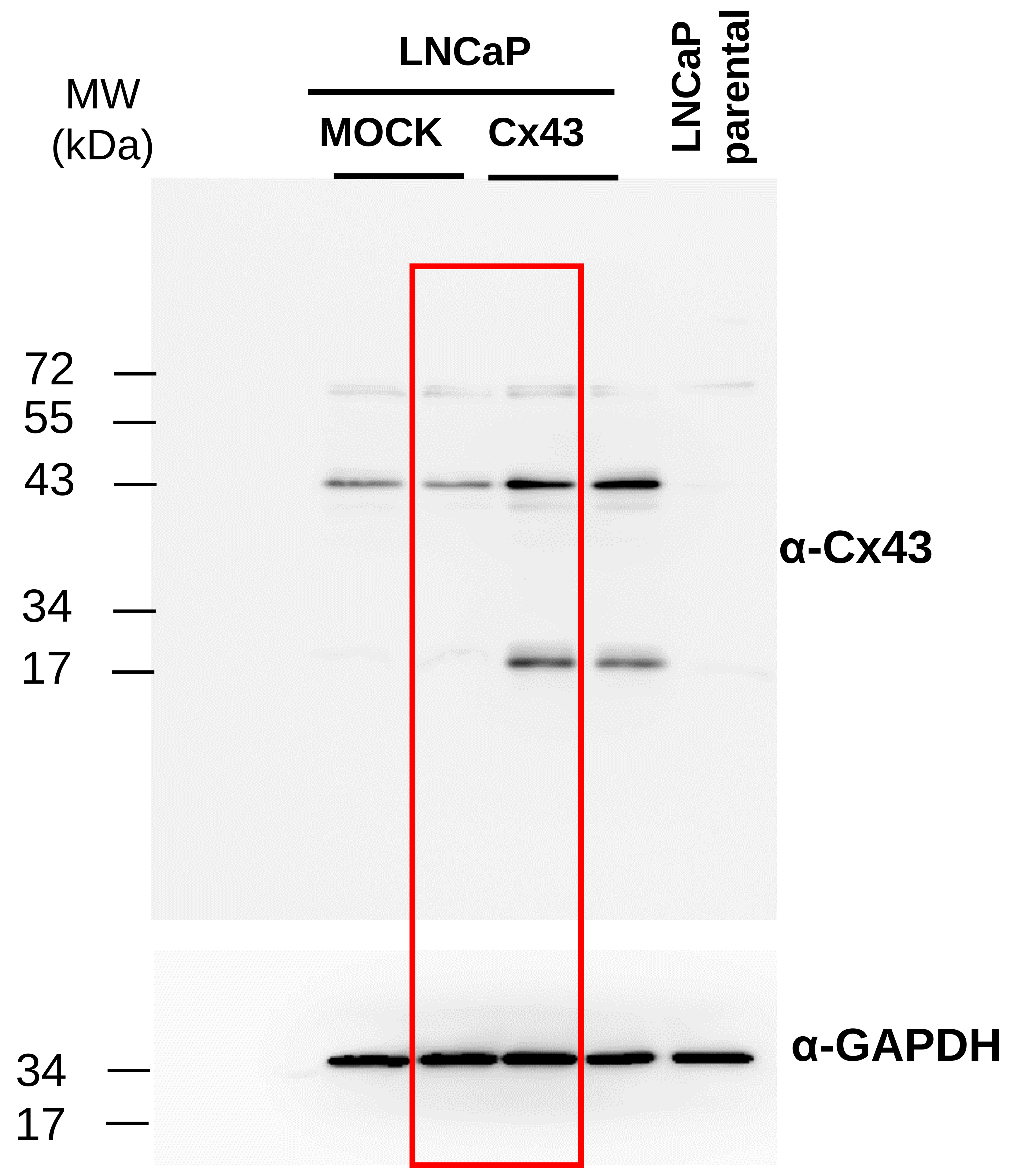

Uncropped from Figure 2C

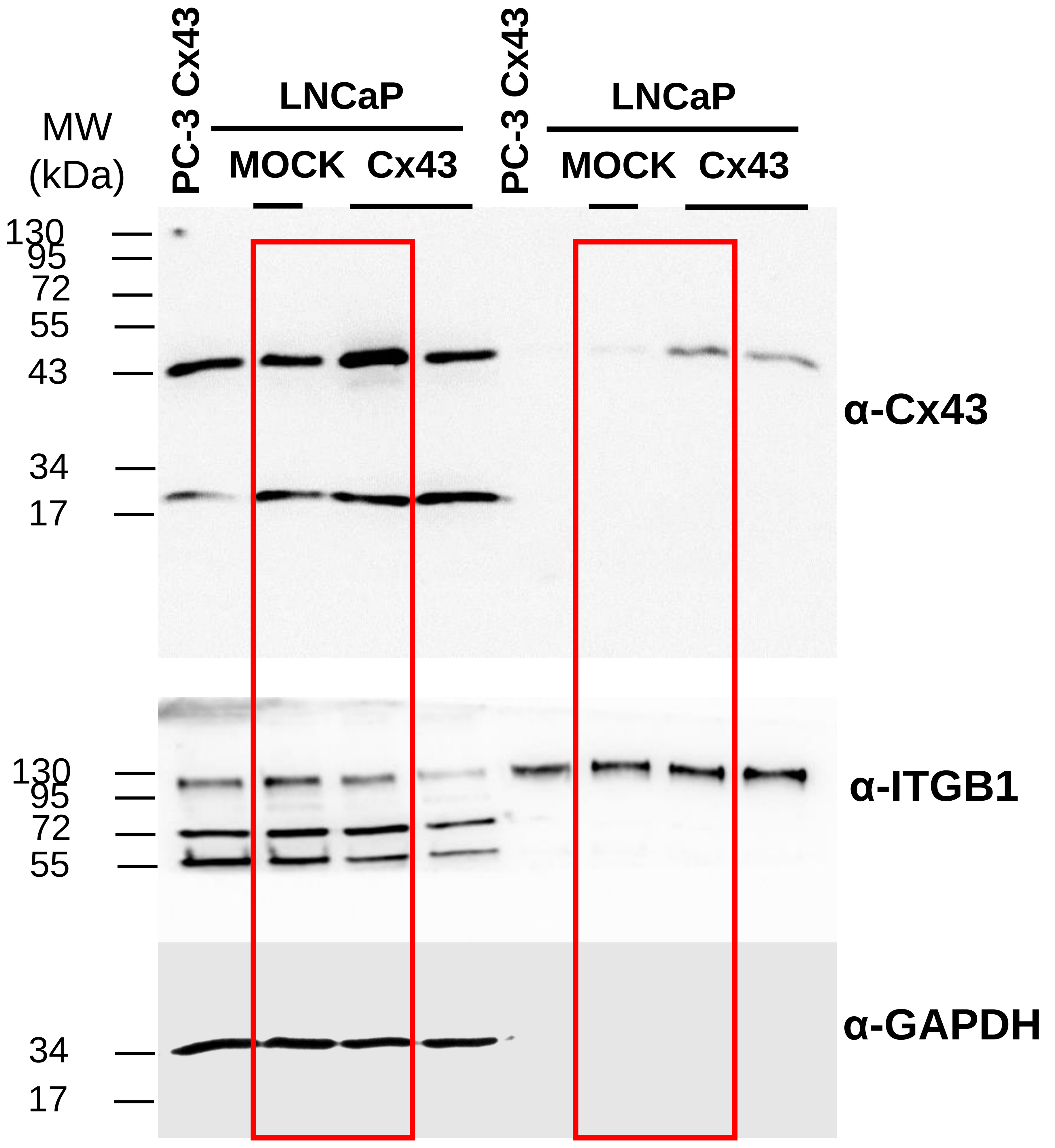

Uncropped from Figure 2D

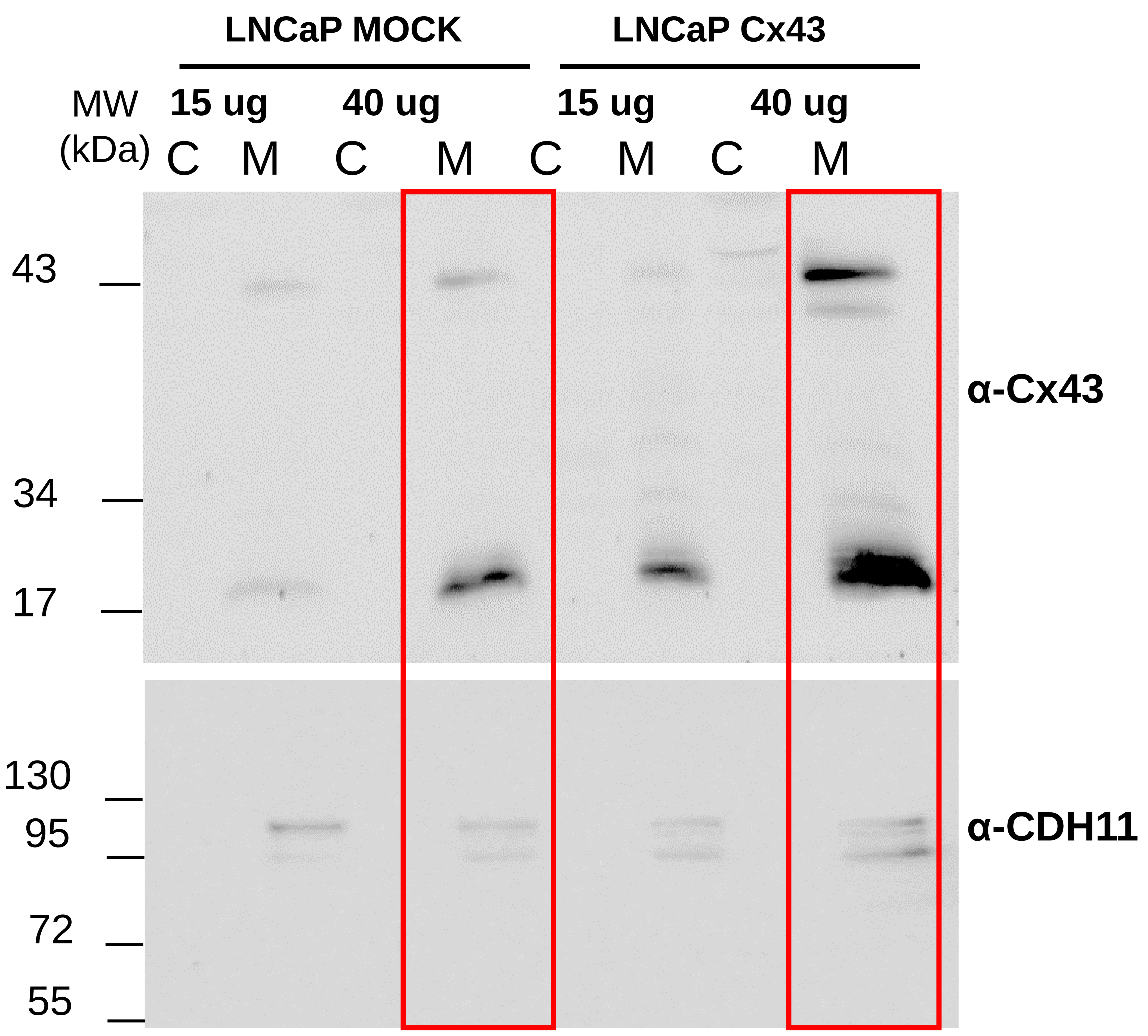

C: cytosol fraction
M: Membrane fraction

Uncropped from Figure 5A

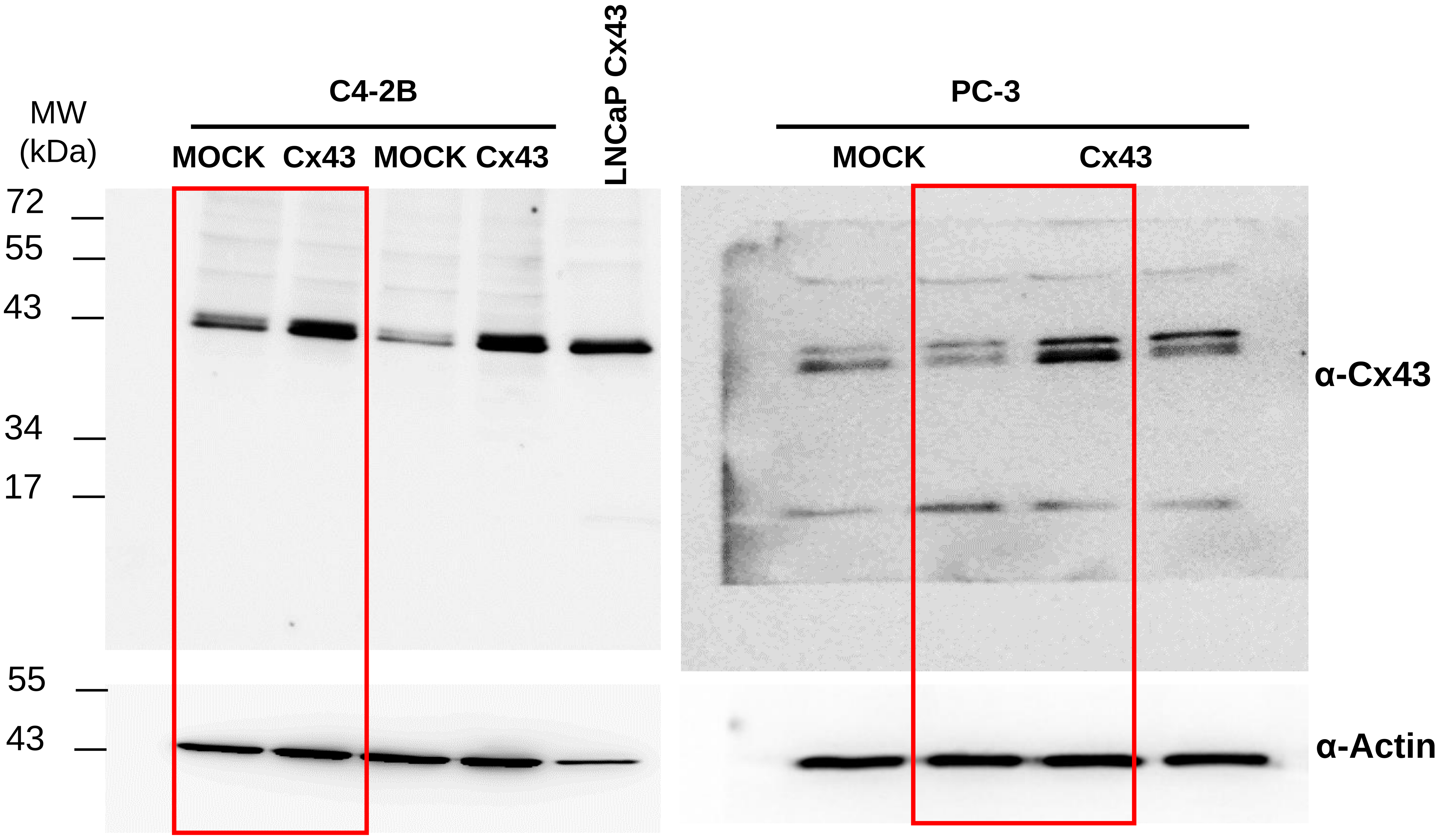

Uncropped from Figure 7A

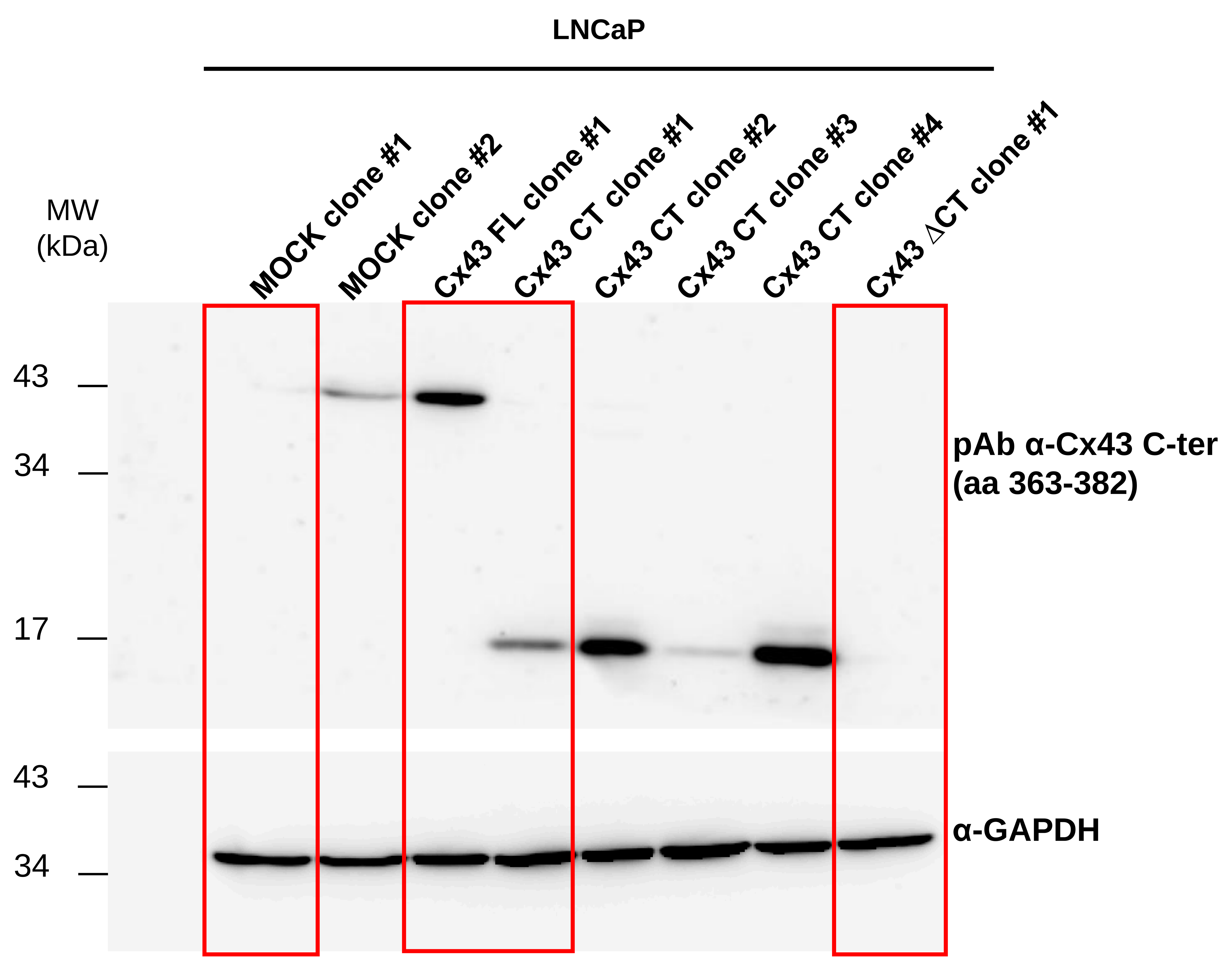

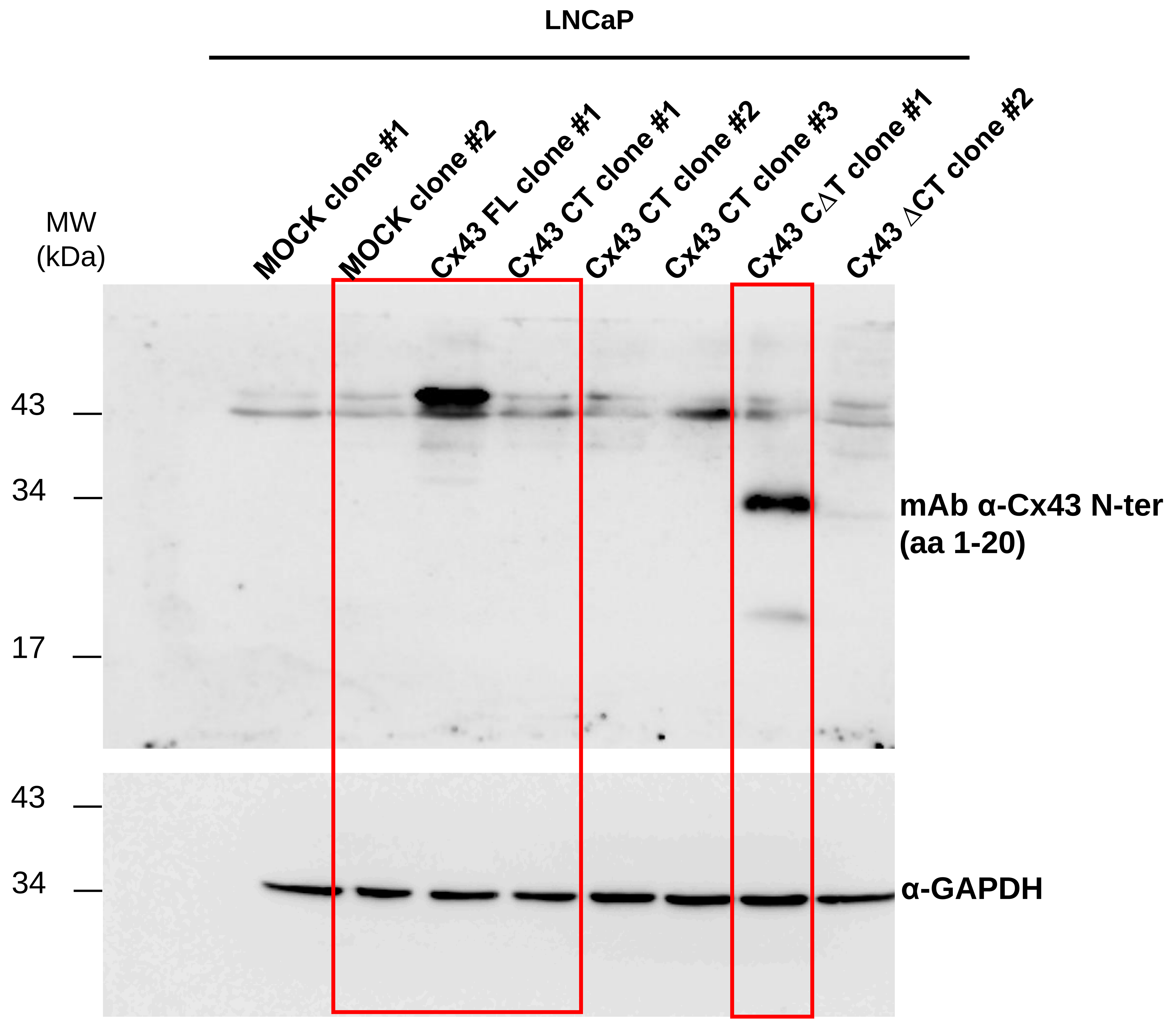

Uncropped from Figure 8B

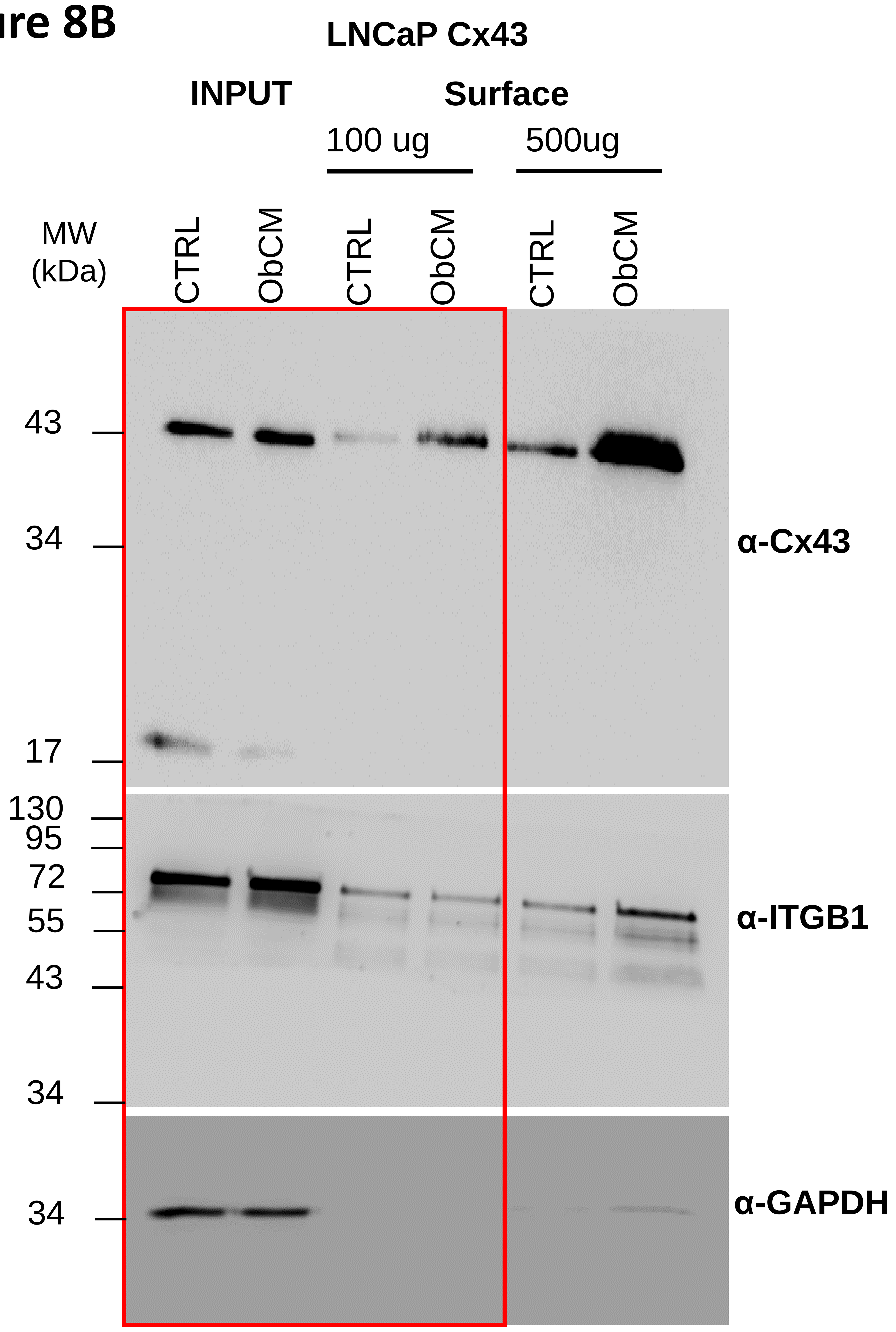

Uncropped from Figure 8E

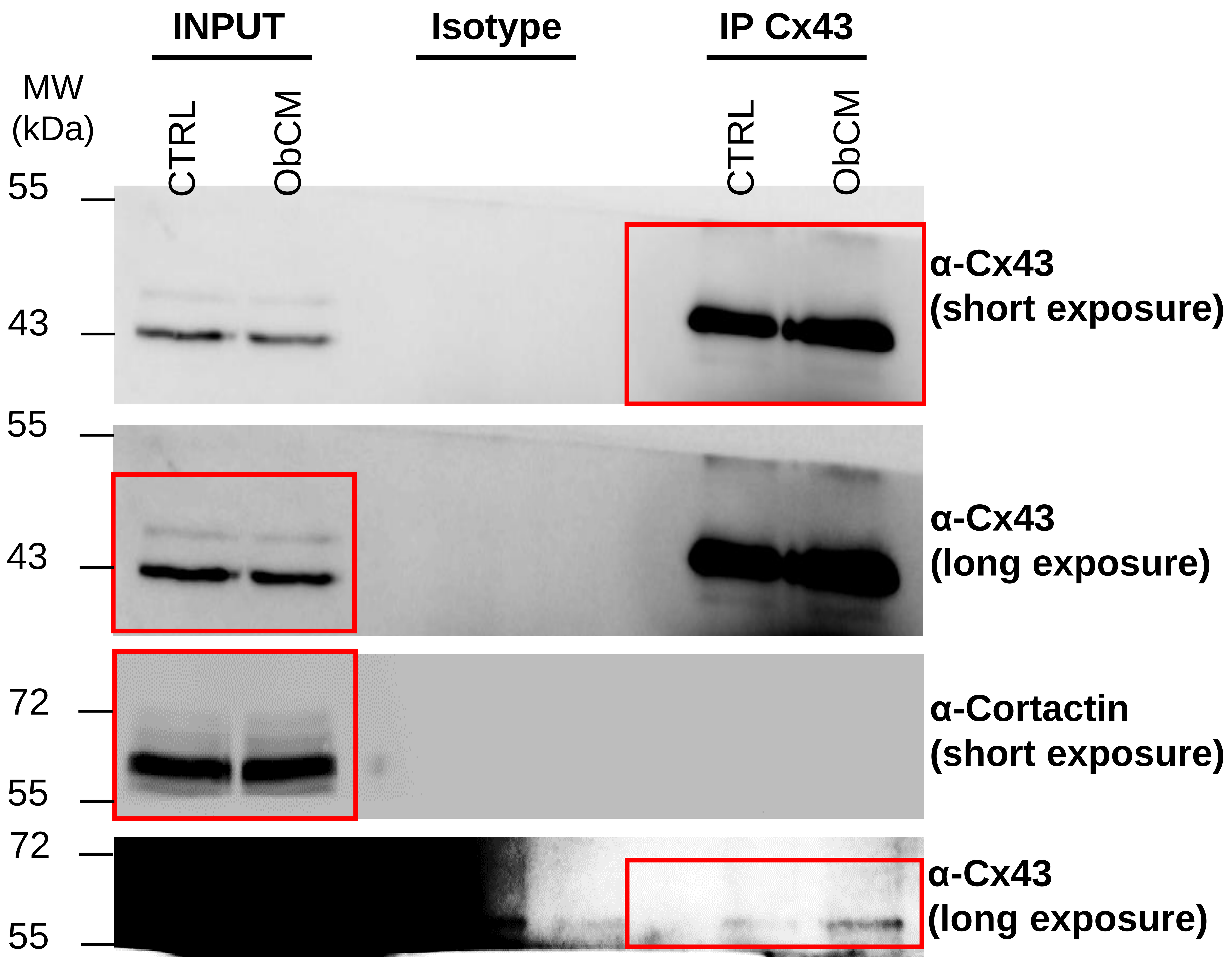

Uncropped from Figure 9A

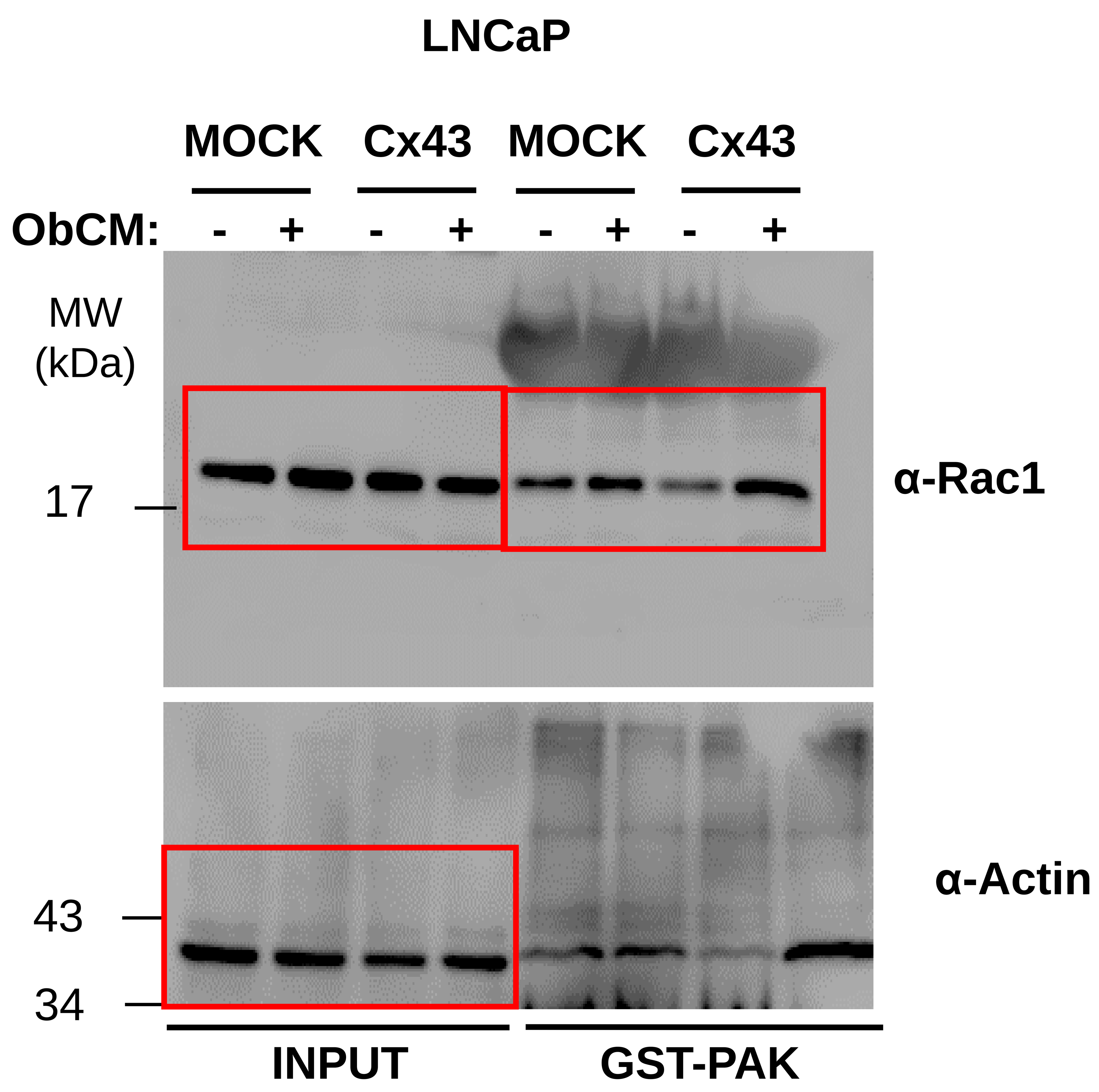

Uncropped from Figure 9C

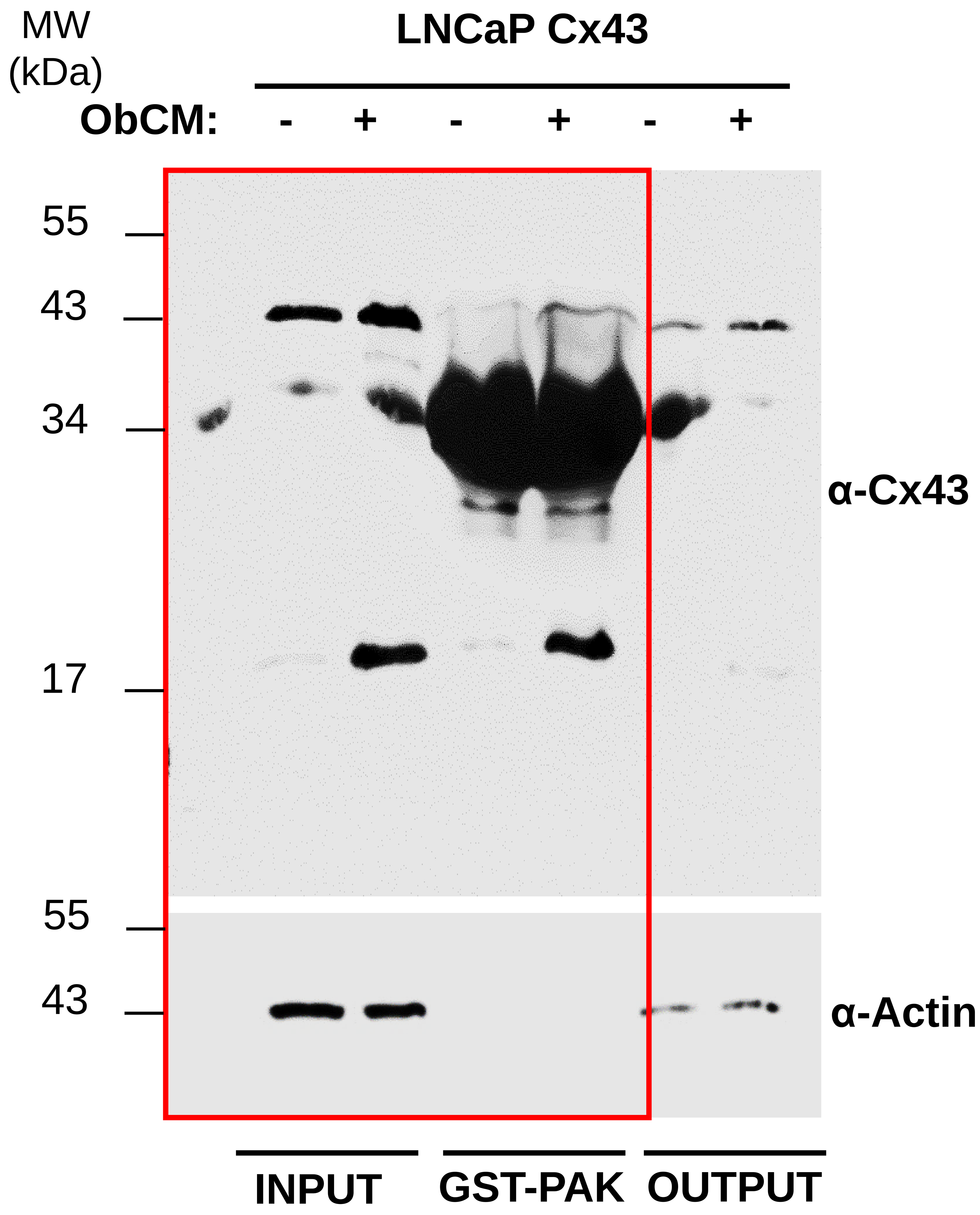

Uncropped from Figure 9B

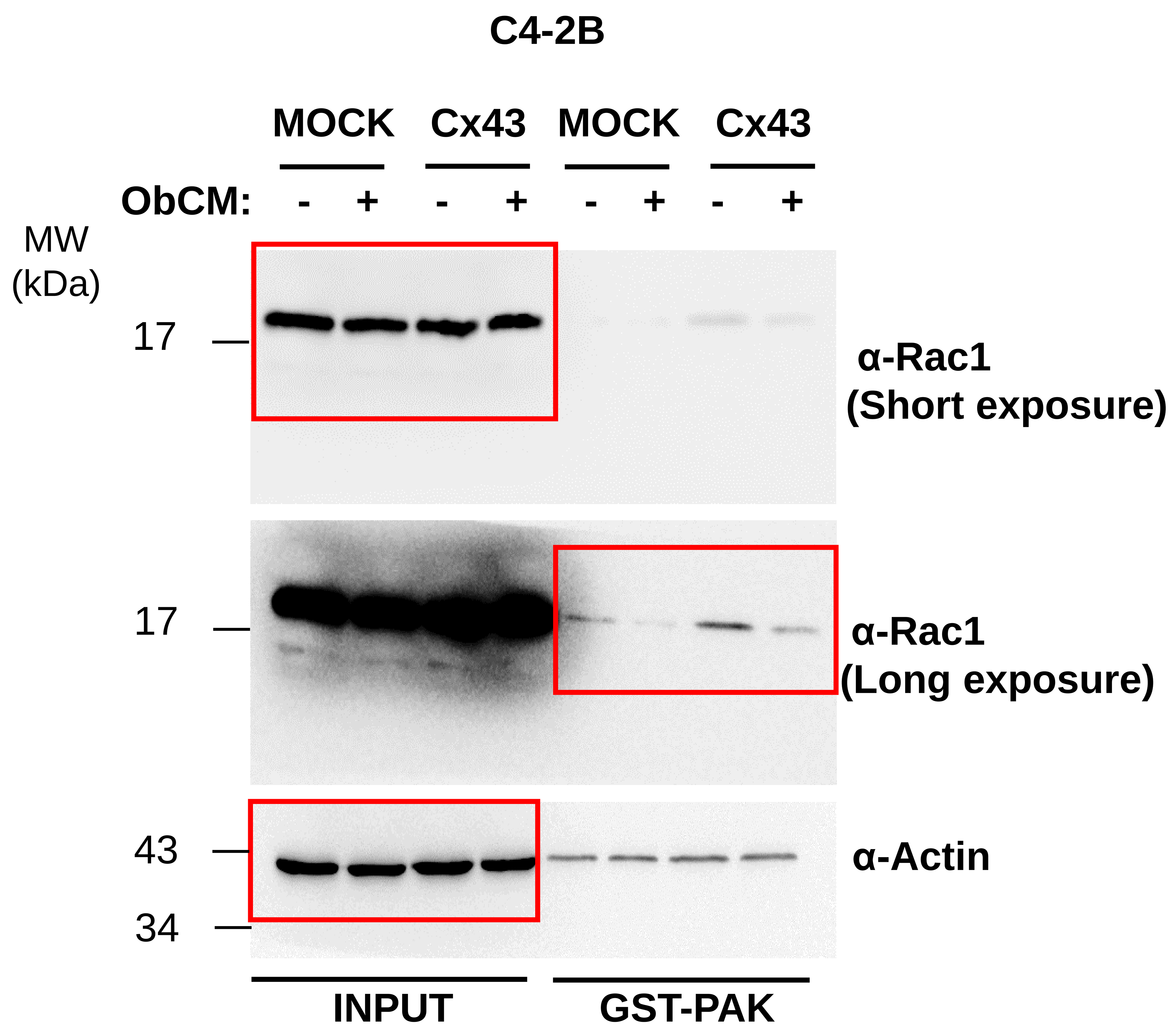

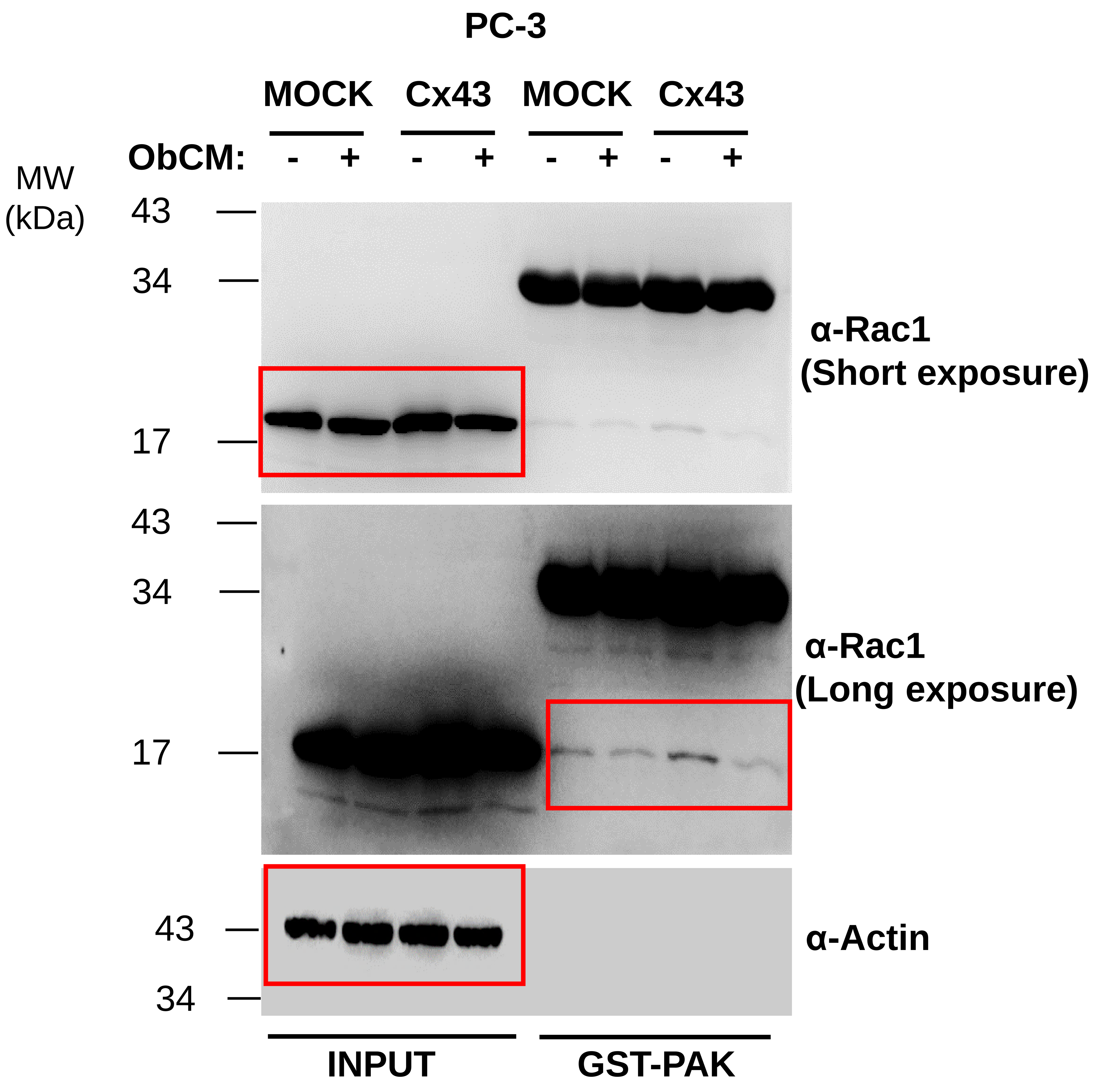

Uncropped from Figure S7

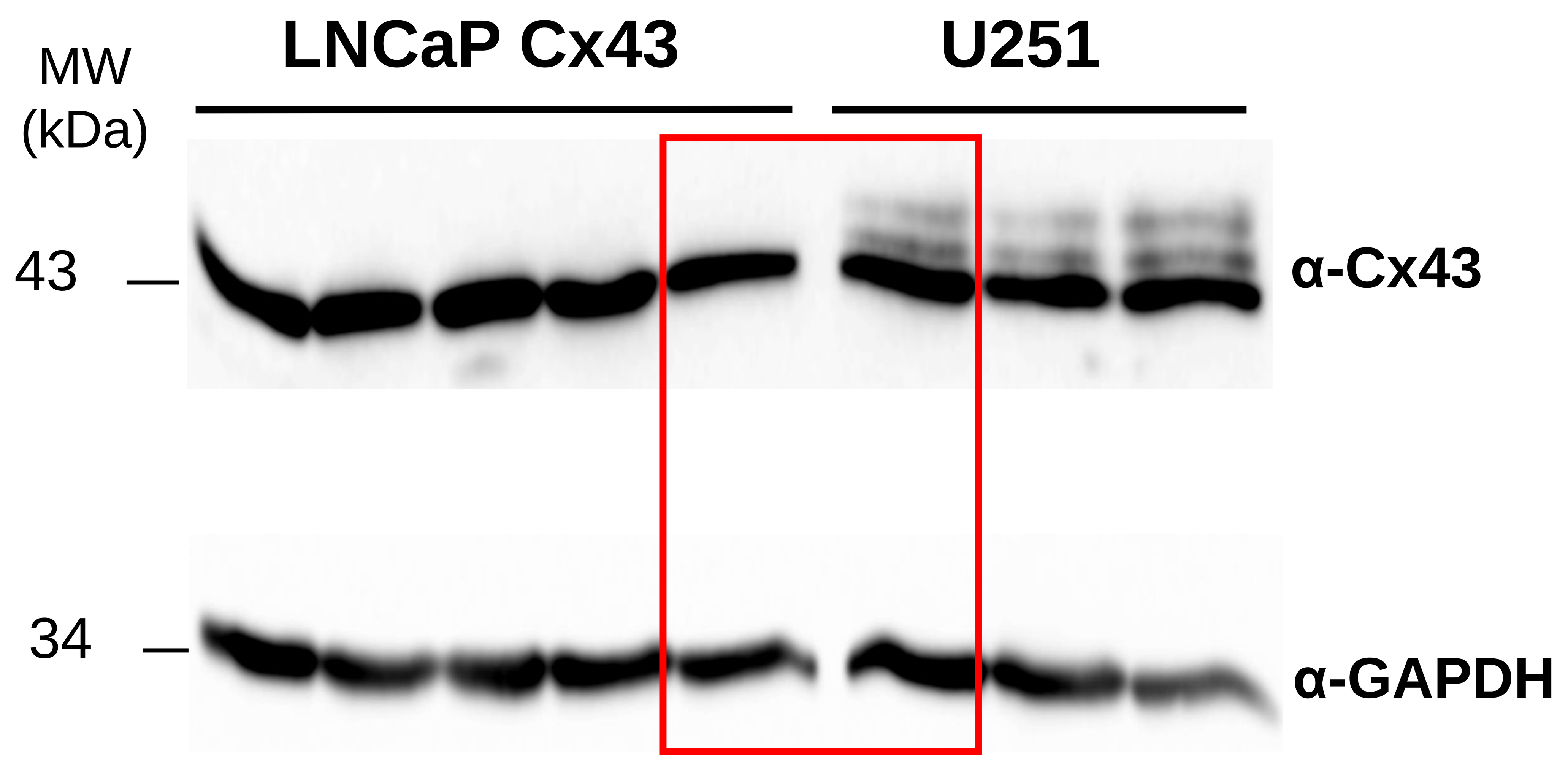

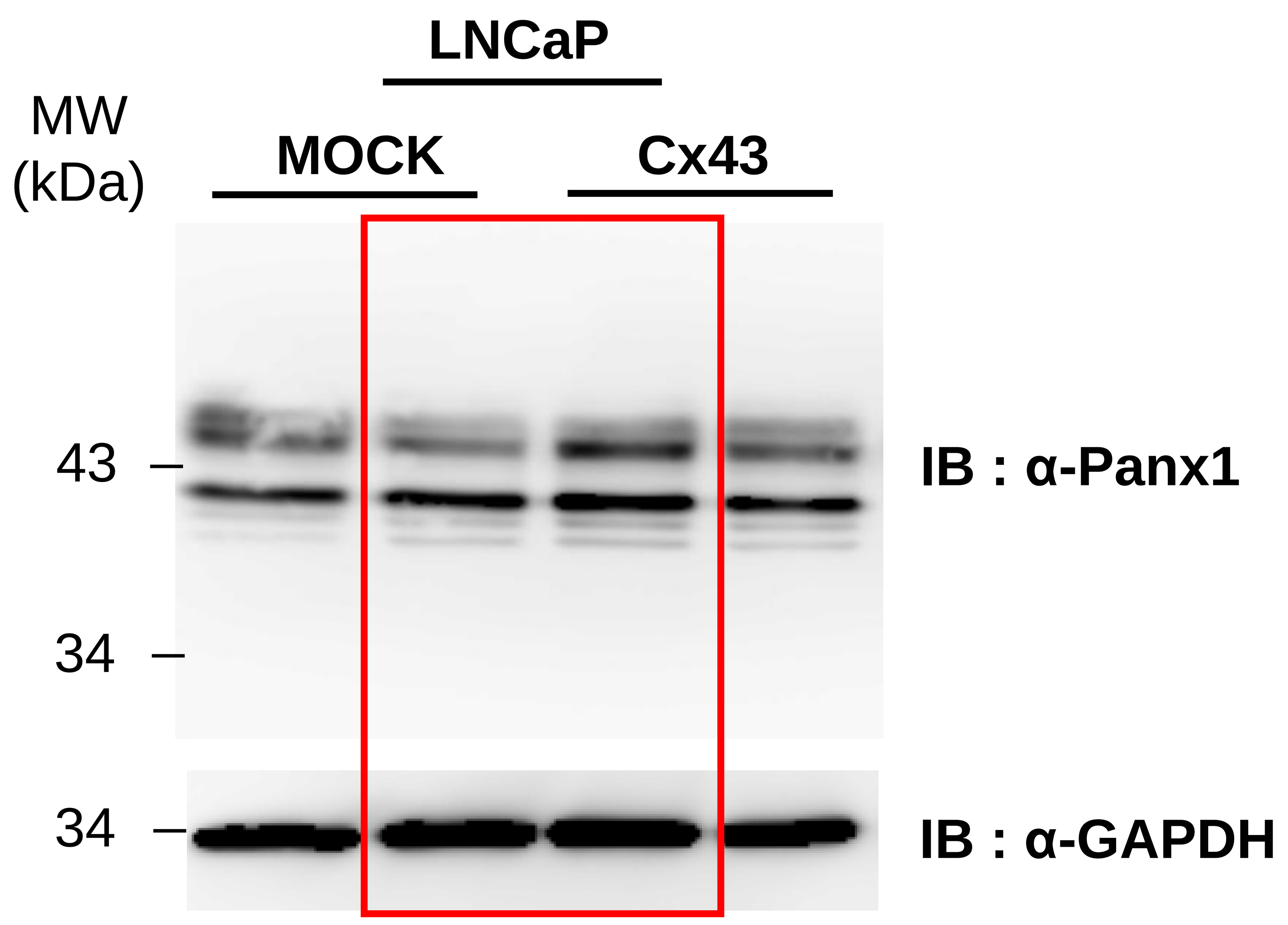

Supplement: Supplementary file 1 [file cancers-12-03013-s001.pdf]
